# A Warburg effect targeting vector designed to increase the uptake of compounds by cancer cells demonstrates glucose and hypoxia dependent uptake

**DOI:** 10.1371/journal.pone.0217712

**Published:** 2019-07-15

**Authors:** Alexandra Glenister, Michela I. Simone, Trevor W. Hambley

**Affiliations:** 1 School of Chemistry, University of Sydney, Camperdown, New South Wales, Australia; 2 Discipline of Chemistry, Priority Research Centre for Chemical Biology & Clinical Pharmacology, University of Newcastle, Callaghan, New South Wales, Australia; Okayama University, JAPAN

## Abstract

Glycoconjugation to target the Warburg effect provides the potential to enhance selective uptake of anticancer or imaging agents by cancer cells. A Warburg effect targeting group, rationally designed to facilitate uptake by glucose transporters and promote cellular accumulation due to phosphorylation by hexokinase (HK), has been synthesised. This targeting group, the C2 modified glucose analogue 2-(2-[2-(2-aminoethoxy)ethoxy]ethoxy)-D-glucose, has been conjugated to the fluorophore nitrobenzoxadiazole to evaluate its effect on uptake and accumulation in cancer cells. The targeting vector has demonstrated inhibition of glucose phosphorylation by HK, indicating its interaction with the enzyme and thereby confirming the potential to facilitate an intracellular trapping mechanism for compounds it is conjugated with. The cellular uptake of the fluorescent analogue is dependent on the glucose concentration and is so to a greater extent than is that of the widely used fluorescent glucose analogue, 2-NBDG. It also demonstrates selective uptake in the hypoxic regions of 3D spheroid tumour models whereas 2-NBDG is distributed primarily through the normoxic regions of the spheroid. The increased selectivity is consistent with the blocking of alternative uptake pathways.

## Introduction

Many of the currently used, clinically-approved anticancer agents have severe side effects resulting from high systemic toxicities, due to their lack of selectivity towards cancerous cells.[1, 2] To improve the efficacy of anticancer agents it is necessary to develop targeted treatments that enable enhanced uptake of anticancer agents by cancer cells relative to normal cells.[3] Selective targeting requires not only a carrier dependent uptake pathway, but also the blocking of other pathways such as passive diffusion.

The metabolic properties of malignant cells differ significantly from those of normal cells, providing the potential to target cellular metabolism to improve the selectivity of anticancer therapeutics.[4, 5] Many metabolic changes exhibited by cancer cells are identified as requirements for malignant transformation, being necessary adaptations to survive the microenvironments found in solid tumours.[6] Recently strategies to target the metabolic differences of cancer cells have been explored, including conjugation of folate, biotin or glucose to exploit the increased consumption of these nutrients by cancer cells.[5, 7–9] These conjugates are designed to be recognised by specific receptors and be taken up by cancer cells at a higher rate than by normal cells.

The avid consumption of glucose by solid tumours compared to normal tissue was first observed by Otto Warburg in the first half of the 20^th^ century.[10] Warburg reported the abnormal energy metabolism of cancer cells with predominantly glycolysis occurring, rather than oxidative phosphorylation, even in the presence of oxygen.[5, 11] The Warburg effect is characterised by increased glucose transport and rates of glucose phosphorylation, and reduced rates of glucose-6-phosphate dephosphorylation.[12] To facilitate their elevated glucose requirements increased levels of glucose transporters (GLUTs) are observed in many malignant cells, with tumours shown to exhibit up to 12-fold higher GLUT activity than normal cells.[13, 14]

The glucose analogue 2-deoxy-2-(^18^F)fluoro-D-glucose (FDG) is a PET imaging agent that exploits the Warburg effect to visualise tumours and their metastases, and is used in over 90% of cancer related scans.[15] The literature contains many examples of glucose conjugated with anticancer drugs, metal complexes and imaging agents to increase their cancer selective delivery, but there has been little progress of any of these compounds through clinical trials, with many glycoconjugates demonstrating limited advantage compared to aglycones.[16, 17] Studies of various glycoconjugates illustrate that GLUT mediated uptake alone may be insufficient to target cancer cells, although uptake of a glycoconjugate may be increased initially.[18, 19] This may be the result of reduced GLUT activity over time as the cell recognises an increased intracellular glucose concentration due to the presence of the glycoconjugate. Alternatively, compounds may be removed from cells, limiting the effectiveness of the targeted uptake. Phosphorylation by hexokinase (HK) can promote trapping of glucose conjugates and maintain uptake of a glucose analogue to ensure enhanced accumulation over time, as is exploited by FDG.[20] To enable targeting, modifications to glucose must ensure retention of GLUT and HK recognition, to promote uptake and accumulation of the glycoconjugates.

## Rational design of Warburg effect targeting vector

Cellular uptake by GLUTs is substrate specific. D-glucose is one of the main sugar substrates transported into cells by GLUT-1,[21] but it has also been demonstrated to have high affinity with GLUT-2, GLUT-3 and GLUT-4,[13] each of which has been shown to be overexpressed by some malignant cells.[22]

Numerous studies have shown the importance of C2 modification of D-glucose for enhanced GLUT mediated uptake and cellular retention of glycoconjugates,[23–25] with the addition of bulky substituents in this position generally well tolerated.[16]

To enable trapping and promote accumulation of a compound, phosphorylation of the glucose analogue is required. Hexokinase inhibition studies of C2-glucosyl-linker functionalised Re complexes with different linker lengths found long linker lengths, of 9 atoms, to result in stronger binding with HK than did short linkers.[26] Use of long alkyl chains as linkers has been demonstrated to reduce the solubility of glucose Re tricarbonyl complexes in aqueous conditions.[26, 27] A poly(ethylene glycol) linker was found to enhance the affinity for HK, compared to the alkyl derivative, and to improve water solubility.[26] It is also expected to reduce lipophilicity and therefore uptake by passive diffusion.

**Fig 1:**
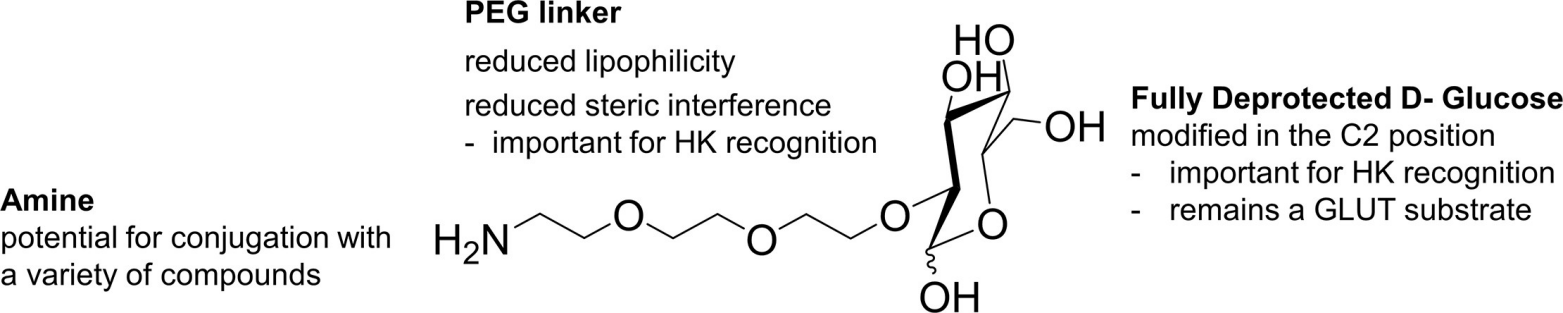
Rationally designed Warburg effect targeting vector.

To fully exploit the potential of targeting the Warburg effect, a glucose analogue rationally designed to facilitate uptake by GLUTs, reduce uptake by other pathways, and be retained in cells due to phosphorylation by HK, has been synthesised (Fig 1). This targeting vector has been designed to be conjugated to various compounds, with the potential to enable increased tumour selective delivery and accumulation of the anticancer prodrugs or imaging agents.

## Experimental procedures

### General

The specific optical rotation of compounds ([α]) was measured using an Optical Activity Limited AA65 Automatic Polarimeter, analytical version (589 nm), with a path length of 1.0 dm, with concentrations (c) quoted in g 100 ml^-1^. IR spectra were collected using UATR Two, PerkinElmer Spectrum Two FT-IR spectrometer over the range 4000–400 cm^-1^. Low resolution ESI mass spectrometry (LRMS) was performed using a Bruker amaZon SL ion trap mass spectrometer. High resolution ESI mass spectra (HRMS) were collected on a Bruker FTICR mass spectrometer. ^1^H and ^13^C NMR spectra were obtained at 300 K on a Bruker 400 MHz or 500 MHz spectrometer.

#### Hexokinase inhibition assay

The ability of compounds to inhibit glucose phosphorylation by hexokinase was tested using glucose (HK) assay kits (Sigma), containing a glucose (HK) assay reagent, reconstituted with 20 mL H_2_O, and 1 mg mL^-1^ glucose standard solution.

5 mM aqueous stock solutions of each compound to be tested were freshly prepared. The assay reagent (280 μL) was combined with glucose standard solution (10 μL) and a volume of compound stock solution, and the total volume made up to 700 μL with H_2_O in a 1 cm pathlength 700 μL quartz fluorescence cuvette (Thorlabs). For each test compound different concentrations were examined by adding known volumes of the stock solution to the assay solution. UV-visible spectrometry was performed on a Cary 60 UV-Visible Spectrophotometer at room temperature, recording absorbance at 340 nm every 2 s for 3 min. Initial rates of reaction (*V*_*i*_) for glucose phosphorylation were determined from the gradient of a plot of absorbance at 340 nm against time.

A control experiment, to calculate the initial rate of glucose phosphorylation in the absence of the compounds, was conducted for each batch of the HK assay reagent used. The initial rate of glucose phosphorylation in the presence of our compounds relative to the control were calculated. These results were plotted against the concentration of compound to show any relationship between compound concentration and rate of HK glucose phosphorylation.

### Cell culture

DLD-1 human colon carcinoma cells were maintained as monolayers in low glucose DMEM supplemented with 10% FBS. The cells were incubated in a humidified environment at 37°C with 5% (v/v) CO_2_, and sub-cultured using trypsin to detach cells.

Confocal imaging of monolayer and spheroid tumour models were performed on a Leica SP5 II microscope, and images were analysed using LAS AF Lite. A heated stage was used to maintain the temperature at 37°C during live cell imaging. The samples were excited with 488 nm light, and the emission wavelengths collected between 530–650 nm.

#### Preparation of monolayer samples for determining glucose dependent uptake of compounds by confocal fluorescence microscopy

1 x 10^5^ cells in 2 mL supplemented low glucose DMEM were plated onto glass-bottom dishes (MatTek) and allowed to adhere overnight. The medium was replaced with glucose free DMEM supplemented with 10% FBS and 2% glutamine. The required volume of glucose solution (200 g L^-1^), to give concentrations from 0 to 8000 mg L^-1^, was added and cells were treated with 50 μM compound for 2 h.

After incubation, the medium was removed and the cells were washed with PBS (2 x 1 mL). Cells were imaged in FluoroBrite DMEM (2 mL) supplemented with 10% FBS and 2% glutamine.

A HCX PL APO 63x 1.2 water objective was used to image monolayer cells. At least 2 dishes for each condition were imaged on each occasion, and the experiment was repeated 2 times.

#### Preparation of spheroid samples for confocal fluorescence imaging

Spheroids were formed by plating 100 μL of a 1 x 10^5^ cells mL^-1^ single cell suspension into each well of an ultra-low cluster, round bottom ultra-low attachment 96-well plate. After allowing the spheroids to aggregate for 3 days, 100 μL of a 100 μM solution of compound in supplemented cell culture medium was added to each well containing a spheroid, to give a final concentration of 50 μM. Following incubation for 4 h, spheroids were collected, the medium removed and the spheroids washed with PBS (2 x 2 mL). Spheroids were suspended in supplemented FluoroBrite DMEM (2 mL), and transferred into a glass-bottom dish (MatTek).

### Synthesis

2-[2-(2-azidoethoxy)ethoxy]ethanol was synthesised by a modification of the method of Legeay *et al*.[28] 2-[2-(2-aminoethoxy)ethoxy]ethanol was synthesised by the procedure of Liu *et al*.[29] 2-[2-(2-azidoethoxy)ethoxy]ethyl mesylate was synthesised by a modification of the procedure of Sakamoto *et al*.[30] 2-NBDG was synthesised by the method of He *et al*.,[31] and purified on a Sephadex LH-20 column eluting with H_2_O.

#### 1,2-O-isopropylidene-α-D-glucofuranose (1)

1,2-*O-*isopropylidene-α-D-glucofuranose was prepared by a modification of the method of Yadav *et al*.[32] Iodine (1.47 g, 5.8 mmol, 0.3 eq.) was added to a solution of 1,2:5,6-di-*O*-isopropylidene-α-D-glucofuranose (5.00 g, 19.2 mmol) in MeCN (270 mL). H_2_O (2 mL) was added, and the reaction mixture was stirred at room temperature for 7 h. The reaction was quenched with saturated aqueous Na_2_S_2_O_3_ solution and extracted into EtOAc (5 x 200 mL). The organic layers were dried over anhydrous Na_2_SO_4_, the solvent removed and the crude mixture was purified by column chromatography (silica) using EtOAc/hexane 2:1, EtOAc and EtOAc/MeOH 1:0.01 to give the product as a white powder (3.29 g, 14.9 mmol, 78% yield). Rf 0.23 (EtOAc, silica); ${\left[\alpha \right]}_{D}^{26}$ -18.3° (*c* 1.00, H_2_O) (lit. ${\left[\alpha \right]}_{D}^{22}$ -12.0° (*c* 1.0, H_2_O)[33]).

#### 3,5,6-tri-O-benzyl-1,2-O-isopropylidene-α-D-glucofuranose (2)

Under N_2_, **1** (1.00 g, 4.5 mmol) was dissolved in anhydrous DMF (24 mL). Sodium hydride in a 60% oily dispersion (0.90 g, 22.5 mmol, 5 eq.) was added portionwise with vigorous stirring. After stirring for 30 min, benzyl bromide (2.80 mL, 22.5 mmol, 5 eq.) was added dropwise. The reaction mixture was stirred at room temperature, under N_2_ for 24 h and then treated with H_2_O (150 mL) and neutralised with 1 M hydrochloric acid. The product was extracted into DCM (3 x 150 mL), and the organic layer was dried over Na_2_SO_4_ and the solvent removed. The product was purified by column chromatography (silica) with hexane/EtOAc 8:1 eluent to yield **2** as a light yellow oil (2.01 g, 4.1 mmol, 90% yield).

Rf 0.46 (hexane/EtOAc 2:1, silica); ${\left[\alpha \right]}_{D}^{26}$ -33.8° (*c* 2.03, CHCl_3_) (lit. ${\left[\alpha \right]}_{D}^{20}$ -36° (*c* 1.0, CHCl_3_)[34]; ${\left[\alpha \right]}_{D}^{25}$ -33° (*c* 9.3, CHCl_3_)[35]).

#### Methyl 3,5,6-tri-O-benzyl-α-D-glucofuranoside (3a) and methyl 3,5,6-tri-O-benzyl-β-D-glucofuranoside (3b)

The method of Lee and Perlin[36] was used for the synthesis of methyl 3,5,6-tri-*O-*benzyl-α-D-glucofuranoside and methyl 3,5,6-tri-*O-*benzyl-β-D-glucofuranoside. **2** (1.00 g, 2.04 mmol) was dissolved in MeOH (20 mL) under N_2_. Amberlite IR-120 (H^+^) ion exchange resin (10.25 g) was added, and the reaction was refluxed under N_2_ for 24 h. The reaction mixture was filtered, the filtrate evaporated and the crude mixture separated by column chromatography (silica) with hexane/EtOAc 4:1 and hexane/EtOAc 2:1 to yield *3a* and *3b* as colourless oils (**3a**: 0.34 g, 0.73 mmol, 36% yield; **3b**: 0.31 g, 0.67 mmol, 33% yield).

#### 3a

Rf 0.53 (hexane/EtOAc 2:1, silica); ${\left[\alpha \right]}_{D}^{26}$ +21.7° (*c* 0.40, CHCl_3_) (lit. ${\left[\alpha \right]}_{D}^{28}$ +28.9° (*c* 0.32, CHCl_3_)[37]).

#### 3b

Rf 0.32 (hexane/EtOAc 2:1, silica); ${\left[\alpha \right]}_{D}^{26}$ -58.7° (*c* 0.21, CHCl_3_) (lit. ${\left[\alpha \right]}_{D}^{28}$ -54.9° (*c* 0.25, CHCl_3_.

#### Methyl 2-(2-[2-(2-azidoethoxy)ethoxy]ethoxy)-3,5,6-tri-O-benzyl-α-D-glucofuranoside (4a) and methyl 2-(2-[2-(2-azidoethoxy)ethoxy]ethoxy)-3,5,6-tri-O-benzyl-β-D-glucofuranoside (4b)

**3a** or **3b** (1.55 g, 3.34 mmol) was dissolved in anhydrous DMF (20 mL) under N_2_. Sodium hydride in a 60% oily dispersion (0.31 g, 7.68 mmol, 2.3 eq.) was added portionwise, and the reaction mixture stirred at room temperature for 20 min. 2-[2-(2-azidoethoxy)ethoxy]ethyl methanesulfonate (1.31 g, 5.18 mmol, 1.55 eq.) in anhydrous DMF (1 mL) was added dropwise to the reaction mixture, which was subsequently stirred at 60°C under N_2_ for 1 week. MeOH (20 mL) was added and the mixture stirred for 30 min, before the solvent was removed. The resulting solid was dissolved in EtOAc (60 mL), washed with H_2_O (2 x 50 mL) and dried over Na_2_SO_4_.

#### 4a

The crude mixture was purified by column chromatography (silica) with hexane/EtOAc 4:1 and EtOAc to give the product as a yellow oil (1.61 g, 2.59 mmol, 78% yield). Rf 0.67 (hexane/EtOAc 1:1, silica); ${\left[\alpha \right]}_{D}^{26}$ +33.3° (*c* 0.25, CHCl_3_); LRMS (ESI+): m/z calculated 644.29 ([M+Na]^+^), found 644.13 ([M+Na]^+^).

#### 4b

The crude mixture was purified by column chromatography (silica) with hexane/EtOAc 9:1 and hexane/EtOAc 4:1 to give the product as a pale yellow oil (1.52 g, 2.44 mmol, 73% yield). Rf 0.55 (hexane/EtOAc 1:1, silica); ${\left[\alpha \right]}_{D}^{26}$ -22.2° (*c* 0.36, CHCl_3_); LRMS (ESI+): m/z calculated 644.29 ([M+Na]^+^), found 644.09 ([M+Na]^+^).

#### Methyl 2-(2-[2-(2-aminoethoxy)ethoxy]ethoxy)-3,5,6-tri-O-benzyl-α-D-glucofuranoside (5a) and methyl 2-(2-[2-(2-aminoethoxy)ethoxy]ethoxy)-3,5,6-tri-O-benzyl-β-D-glucofuranoside (5b)

A solution of **4a** or **4b** (1.60 g, 2.57 mmol) in MeOH (25 mL) was stirred under N_2_. Pd(10%)/C (160 mg, 10% w/w) was added and the mixture was stirred under an atmosphere of hydrogen for 2 h at room temperature. The catalyst was removed by filtration through celite and solvent removed from the filtrate to yield the products as a pale-yellow oils (**5a**: 1.47 g, 2.47 mmol, 96% yield; **5b**: 1.50 g, 2.52 mmol, 98% yield).

#### 5a

${\left[\alpha \right]}_{D}^{26}$ +76.7° (*c* 0.20, CHCl_3_); LRMS (ESI+): m/z calculated 596.32 ([M+H]^+^), found 596.00 ([M+H]^+^).

#### 5b

${\left[\alpha \right]}_{D}^{26}$ -20.9° (*c* 0.86, CHCl_3_); LRMS (ESI+): m/z calculated 596.32 ([M+H]^+^), found 596.28 ([M+H]^+^).

#### Methyl 2-(2-[2-(2-(tert-butoxycarboxamido)ethoxy)ethoxy] ethoxy)-3,5,6-tri-O-benzyl-α-D-glucofuranoside (6a) and methyl 2-(2-[2-(2-(tert-butoxycarboxamido)ethoxy)ethoxy] ethoxy)-3,5,6-tri-O-benzyl-β-D-glucofuranoside (6b)

**5a** or **5b** (0.70 g, 1.18 mmol) was dissolved in MeCN (17.5 mL). Et_3_N (0.17 mL, 1.18 mmol, 1 eq.) and di-*tert*-butyl dicarbonate (0.26 g, 1.18 mmol, 1 eq.) were added and the reaction mixture stirred for 5 h at room temperature. The solvent was removed and the resulting solid partitioned between H_2_O (10 mL) and EtOAc (10 mL). The EtOAc layer was collected and washed with H_2_O (7 mL). The organic layer was dried over Na_2_SO_4_ and the solvent removed to give the products as yellow oils (**6a**: 0.62 g, 0.89 mmol, 75% yield; **6b**: 0.58 g, 0.83 mmol, 70% yield).

#### 6a

Rf 0.85 (EtOAc, silica); ${\left[\alpha \right]}_{D}^{26}$ +34.2° (*c* 0.37, CHCl_3_); LRMS (ESI+): m/z calculated 718.36 ([M+Na]^+^), found 718.00 ([M+Na]^+^).

#### 6b

Rf 0.82 (EtOAc, silica); ${\left[\alpha \right]}_{D}^{26}$ -17.5° (*c* 0.85, CHCl_3_); LRMS (ESI+): m/z calculated 718.36 ([M+Na]^+^), found 718.37 ([M+Na]^+^).

#### Methyl 2-(2-[2-(2-(tert-butoxycarboxamido)ethoxy)ethoxy] ethoxy)-α-D-glucofuranoside (7a) and methyl 2-(2-[2-(2-(tert-butoxycarboxamido)ethoxy)ethoxy] ethoxy)-β-D-glucofuranoside (7b)

A 9 mM solution of **6a** or **6b** in EtOH was passed through a ThalesNano H-Cube P flow hydrogenation reactor at 0.3 mL min^-1^. Benzyl ether hydrogenolysis was achieved using a Pd(10%)/C CatCart at 80 bar and 60°C. The product solution was evaporated to dryness to yield the product as a colourless oil, without any further purification (quantitative yield).

#### 7a

${\left[\alpha \right]}_{D}^{26}$ +64.0° (*c* 0.50, H_2_O); LRMS (ESI+): m/z calculated 426.23 ([M+H]^+^), found 426.51 ([M+H]^+^).

#### 7b

${\left[\alpha \right]}_{D}^{26}$ -33.3° (*c* 0.24, H_2_O); LRMS (ESI+): m/z calculated 448.22 ([M+Na]^+^), found 448.21 ([M+Na]^+^).

#### Hydrochloride salt of 2-(2-[2-(2-aminoethoxy)ethoxy]ethoxy)-D-glucose (8)

**7a** or **7b** (0.20 g, 0.47 mmol) was heated at reflux in 0.5 M hydrochloric acid (12 mL) for 24 h. The solvent was removed, and the product was obtained as a beige solid after freeze-drying (0.15 g, 0.47 mmol, quantitative yield). ${\left[\alpha \right]}_{D}^{26}$ +43.9° (*c* 0.94, H_2_O); LRMS (ESI+): m/z calculated 312.17 ([M+H]^+^) and 334.15 ([M+Na]^+^), found 312.14 ([M+H]^+^) and 334.13 ([M+Na]^+^); HRMS (ESI+): m/z calculated 312.16559 ([M+H]^+^) for C_12_H_26_O_8_N, found 312.16529 ([M+H]^+^).

#### 2-(2-[2-(2-(N-(7-Nitrobenz-2-oxa-1,3-diazol-4-yl))aminoethoxy)ethoxy]ethoxy)-D-glucose (9)

**8** (90 mg, 0.259 mmol, 1.1 eq.) was dissolved in MeOH (3 mL) and Et_3_N (99 μL, 0.706 mmol, 3 eq.) was added. After stirring at 30°C for 1 h, NBD-Cl (47 mg, 0.235 mmol, 1 eq.) was added. The reaction was stirred overnight in the dark and at 30°C. Solvent was removed and the resulting solid was dissolved in EtOAc (5 mL). Insoluble material was removed by filtration, and the filtrate was dried *in vacuo*. The resulting solid was dissolved in H_2_O (5 mL) and purified on a sephadex LH-20 column, eluting with H_2_O to give the product as an orange solid (35 mg, 0.074 mmol, 29% yield).

LRMS (ESI+): m/z calculated 497.12 ([M+Na]^+^), found 497.15 ([M+Na]^+^); HRMS (ESI+): m/z calculated 497.14903 ([M+Na]^+^) for C_18_H_26_N_4_O_11_Na, found 497.14954 ([M+Na]^+^).

#### ((N-(7-Nitrobenz-2-oxa-1,3-diazol-4-yl)aminoethoxy)ethoxy)ethanol (10)

A solution of 2-[2-(2-aminoethoxy)ethoxy]ethanol (0.41 g, 2.75 mmol, 1.1 eq.) and Et_3_N (660 μL, 4.75 mmol, 1.9 eq.) in methanol (15 mL) was stirred at 30°C for 1 h. NBD-Cl (0.5 g, 2.50 mmol, 1 eq.) was added and the reaction was stirred in the dark for 16 h at 30°C. Insoluble material was removed by filtration, and the filtrate was dried. The resulting solid was dissolved in H_2_O (5 mL) and purified on a sephadex LH-20 column, eluting with H_2_O to give the product as an orange solid (0.33 g, 1.05 mmol, 42% yield).

LRMS (ESI-): m/z calculated 311.10 ([M-H]^-^), found 311.18 ([M-H]^-^), (ESI+): m/z calculated 335.27 ([M+Na]^+^), found 335.09 ([M+Na]^+^); HRMS (ESI+): m/z calculated 335.09621 ([M+Na]^+^) for C_12_H_16_N_4_O_6_Na, found 335.09650 ([M+Na]^+^).

## Results and discussion

### Discussion of the synthesis of the Warburg effect targeting vector

**Fig 2:**
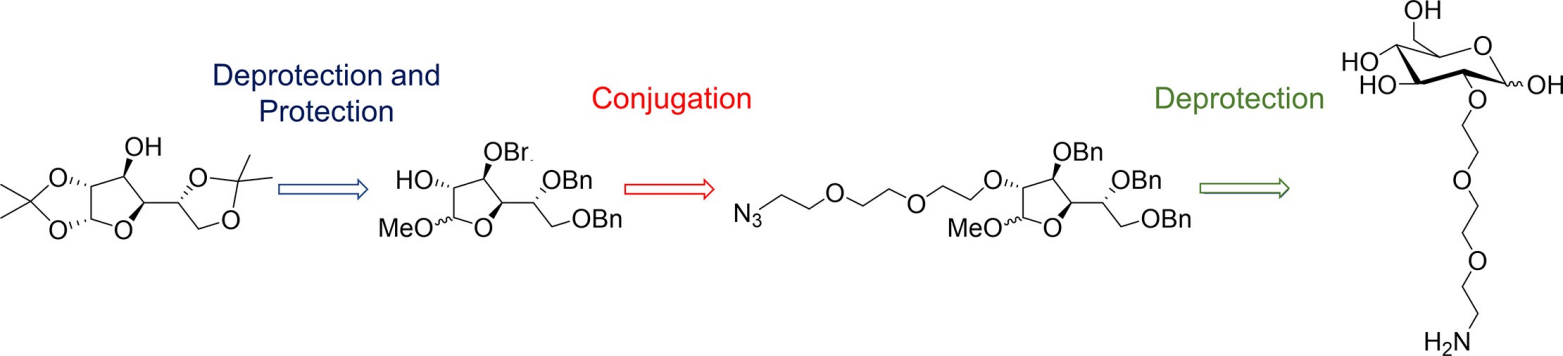
Synthetic route to novel C2 modified glucose analogue for GLUT-1 and HK recognition (compound 8). **a**: I_2_, H_2_O, MeCN, 78% yield; **b**: NaH, benzyl bromide, DMF, 90% yield; **c**: Amberlite IR-120(H^+^), MeOH, reflux, yield–α 36% and β 33%; **d**: NaH, DMF, 60 ºC, yield–α 78% and β 73%; **e**: Pd(10%)/C, H_2_(g), MeOH, yield–α 96% and β 98%; **f**: Boc anhydride, Et_3_N, MeCN, yield–α 75% and β 70%; **g**: Pd(10%)/C CatCart, H_2_(g), 80 Bar, 60°C, quantitative yield; **h**: 0.5 M HCl (aq.), reflux, quantitative yield.

To yield the target glucose analogue 2-(2-[2-(2-aminoethoxy)ethoxy]ethoxy)-D-glucose from 1,2:5,6-di-*O*-isopropylidene-α-D-glucofuranose, protection of all positions other than the C2 hydroxyl group was necessary, followed by coupling of the linker at the C2 position and finally removal of all protecting groups (Fig 2). Maintaining the stereochemistry of the starting compound was essential to ensure synthesis of a D-glucose analogue, required for GLUT and HK recognition.

#### Synthesis of a glucose analogue with only the C2 hydroxyl group deprotected

The initial synthetic steps involve protection of the hydroxyl groups in the C1, C3, C4, C5 and C6 positions of glucose. Using existing literature syntheses, a synthetic route to obtain D-glucose analogues with a hydroxyl group in only the C2 position was devised. The α- and β-anomers (compounds **3a** and **3b**) were isolated by silica column chromatography and used separately in further syntheses for ease of purification and optimisation of subsequent reactions. Upon full deprotection of the glucose analogue, both α- and β-anomers give the same product as the compounds will interconvert in solution.

#### Modification at the C2 position

Conjugation of a linker at the C2 position of **3a** and **3b** by ether synthesis proceeds via an S_N_2 reaction. S_N_2 reactions result in inversion of stereochemistry at the carbon centre of substitution, therefore to maintain the stereochemistry of the glucose analogue the C2 position must act as a nucleophile, formed via proton abstraction by a strong base, and a good leaving group must be on the PEG linker. A selection of bases and leaving groups were investigated to optimise the conjugation of a linker to **3a** and **3b**.

The addition of a good leaving group on 2-[2-(2-azidoethoxy)ethoxy]ethanol increases its reactivity, required for an S_N_2 reaction, but also decreases the stability of the PEG compound. The mesylate leaving group was found to be ideal, as it does not degrade before it can undergo the S_N_2 reaction with the glucose analogue. 2-[2-(2-azidoethoxy)ethoxy]ethyl mesylate enabled conjugation of PEG to glucose C2, with good yield at increased temperatures and with reproducible results.

The reactivities of **3a** and **3b** were found to differ. It is hypothesised that the different stereochemistries of the anomers affects the approach of the base for proton abstraction. Steric hindrance present in the β-anomer prevented alkoxide formation by lithium diisopropylamide, therefore the less bulky strong base NaH was used. Optimisation of the number of NaH equivalents used, reaction time and temperature provided conditions that are applicable to both anomers.

#### Deprotecting 4a and 4b

Azides are reduced under the hydrogenation conditions used for benzyl ether cleavage, which provided the possibility for simultaneous amine formation and the removal of benzyl ether protecting groups. However for compounds **4a** and **4b** this was unsuccessful, even at increased temperature and pressure, as reduction of the azide occurred prior to benzyl ether deprotection. Surfraz *et al*. have demonstrated that starting materials with amines poison Pd/C catalysts, affecting reactivity towards OBn protecting groups.[38] The amine produced by azide reduction of **4a** and **4b** poisons the catalyst, preventing cleavage of the benzyl ether groups. This conclusion is supported by the work of Sajiki *et al*., who report the inhibition of Pd/C catalysed OBn deprotection for substrates containing reducible functional groups that give amine products, including N-Cbz and NO_2_.[39]

To prevent poisoning of the Pd catalyst, to enable complete benzyl ether cleavage, protection of the primary amine produced by hydrogenation (compounds **5a** and **5b**) was required. *Tert*-butyloxycarbonyl (Boc) was identified as a suitable protecting group as it withstands hydrogenation conditions, and benzyl ether cleavage by Pd/C catalysed hydrogenolysis of molecules containing Boc functionality has been demonstrated.[39, 40] Additionally Boc can be removed with acid,[41, 42] the same conditions required for methyl glycoside deprotection.[43] Following Boc protection, to give **6a** and **6b**, complete benzyl ether cleavage proceeded via Pd(10%)/C catalysed hydrogenolysis only at elevated temperature and pressure to give **7a** and **7b**.

The final reaction to remove the Boc and methyl glycoside groups by refluxing in 0.5 M hydrochloric acid yielded the targeting vector as the HCl salt (**8**). Methyl glycoside cleavage deprotects the C1 glucose position, enabling interconversion in aqueous solution between the α- and β-anomers. The deprotected C1 and C6 hydroxyl groups provide the ability for this compound to adopt the six-membered pyranose ring structure, which is favoured over the furanose form for D-glucose.[44, 45] The α- and β-anomers of **8** are observed in the ^1^H and ^13^C NMR spectra collected in D_2_O. The C1 protons of the compound are identifiable in the ^1^H NMR spectrum, being downfield from other signals as for α,β-D-glucose,[46] and the significant difference in chemical shift for C1 protons between the anomers of D-glucose,[47] enabled identification of C1 anomeric protons in the ^1^H NMR spectrum of **8**. Integration of these peaks showed that the α- and β-anomers to be present in equal amounts in solution.

### Hexokinase inhibition studies

Once glucose enters cells it is metabolised to pyruvate through the process of glycolysis. Glucose is initially phosphorylated by adenosine triphosphate (ATP) to give to glucose-6-phosphate (G6P) in a reaction catalysed by HK. Phosphorylated C2 modified glucose analogues, such as FDG and 2-NBDG, cannot undergo the structural rearrangement required for subsequent glycolysis steps and the G6P analogue is trapped intracellularly.[48] Targeting vector **8** has been designed to provide this trapping mechanism for its conjugates.

The inhibition of HK glucose phosphorylation by glucosamine hydrochloride, the aglycone 2-[2-(2-aminoethoxy)ethoxy]ethanol and **8** has been investigated using a glucose (HK) assay reagent containing HK, ATP, NAD^+^ and glucose-6-phosphate dehydrogenase (G6PDH). Following the addition of a glucose solution, a series of enzyme catalysed reactions occur that enable quantification of the rate of HK glucose phosphorylation by monitoring the conversion of NAD^+^ to NADH by UV spectroscopy. The glucose (HK) assay reveals whether a compound interacts with HK to inhibit its active site, but does not establish that phosphorylation of the compound occurs.

Glucosamine is a known inhibitor of glucose phosphorylation by HK, being a substrate for the enzyme, it is recognised by the active site and phosphorylated.[49, 50] Therefore the inhibition of HK glucose phosphorylation by glucosamine hydrochloride serves as a useful comparison to the inhibitory capacity of the novel glucose analogue synthesised.

The results (S1 Fig) demonstrate that **8** exhibits inhibition of HK activity, at a level of about 30% of that exhibited by glucosamine, and that no inhibition is observed for the aglycone. These results indicate that the targeting vector (**8**) fulfils the criteria of providing HK recognition, giving rise to the potential to facilitate an intracellular trapping mechanism for its conjugates.

The low inhibition by compound **8** compared to glucosamine is possibly due to the flexibility of the PEG linker preventing strong interactions with HK, and is as observed for glucose analogue ligands reported by Schibli *et al*.[26] However for conjugation to bulky groups, a linker of sufficient length has been demonstrated to be essential for inhibition of HK.[20, 27, 48] Inhibition of HK glucose phosphorylation by Re complexes conjugated to C2 modified glucose analogue ligands, with 9 atom length linkers, has been demonstrated to be enhanced compared to the free ligand.[26] It is possible that the presence of a bulky group in the outer cavity of HK provides stabilising interactions with the enzyme surface, which provides enhanced binding between the active site and the glucose analogue. Therefore **8** has the potential to provide HK interaction and phosphorylation to its conjugates, which is unlikely for glucosamine conjugates.

### Fluorescent analogues

**Fig 3:**
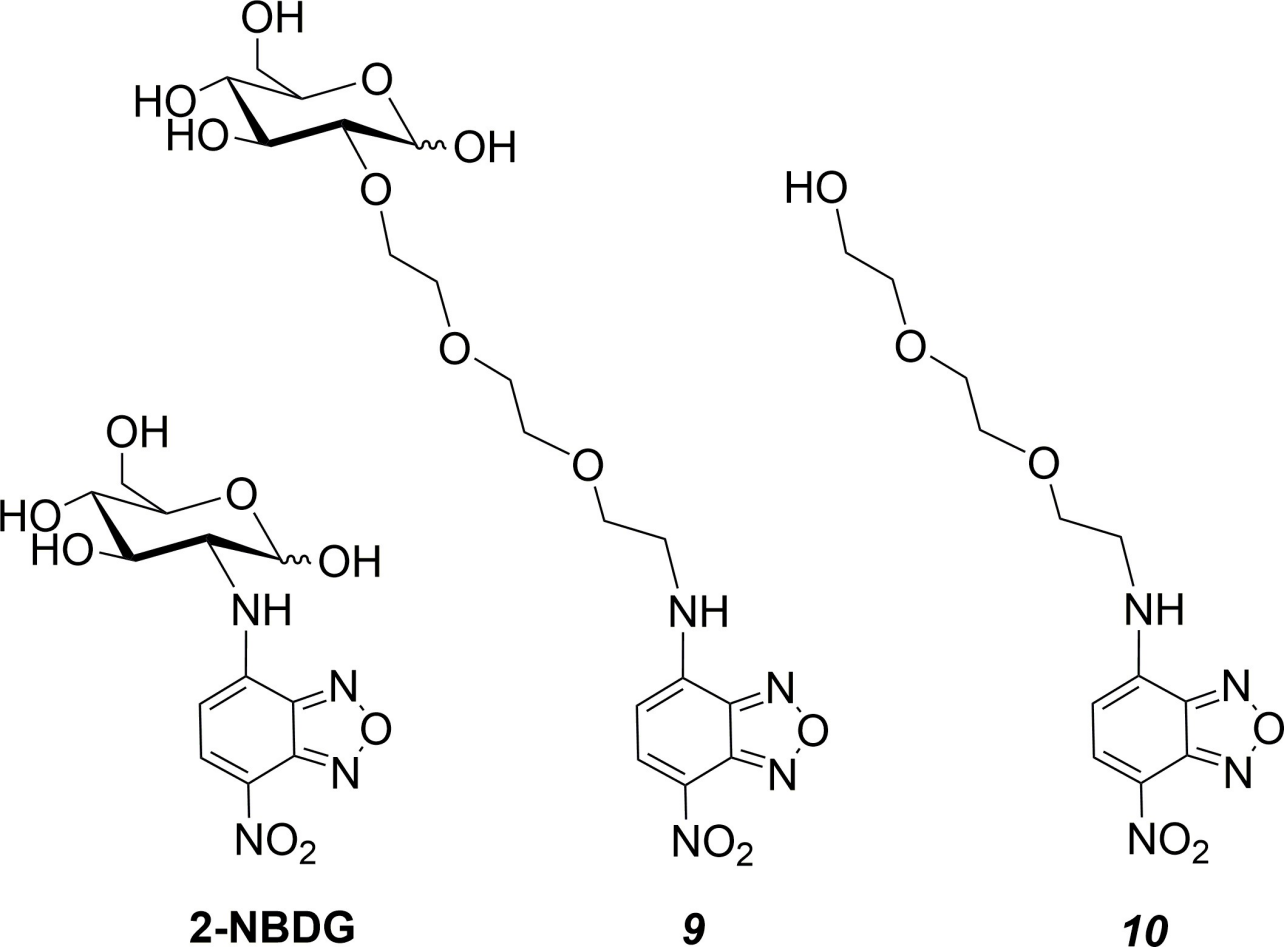
Structures of fluorescent analogues synthesised to investigate Warburg effect targeting *in vitro*.

Glucose uptake by cells *in vitro* can be imaged using the fluorescent glucose analogue 2-NBGD (Fig 3). As for D-glucose, this C2 modified glucose analogue is taken into cells via GLUT transporters and phosphorylated by HK.[23, 51] 2-NBDG fluorescence has been used to quantify glucose uptake in cell monolayers, and 3-dimensional cell aggregates and spheroids.[52, 53] Fluorescent analogues of **8** and the aglycone 2-[2-(2-aminoethoxy)ethoxy]ethanol (Fig 3) were synthesised following modification of the synthesis of 2-NBDG from NBD-Cl,[31] to visualise and compare uptake of these compounds *in vitro* and their fluorescence spectra are shown in S2 Fig. Comparison of the uptake and distribution of **9** with 2-NBDG and the aglycone **10** will enable investigation and evaluation of the Warburg effect targeting of **8**.

#### Glucose dependent uptake in monolayer cell culture

**Fig 4:**
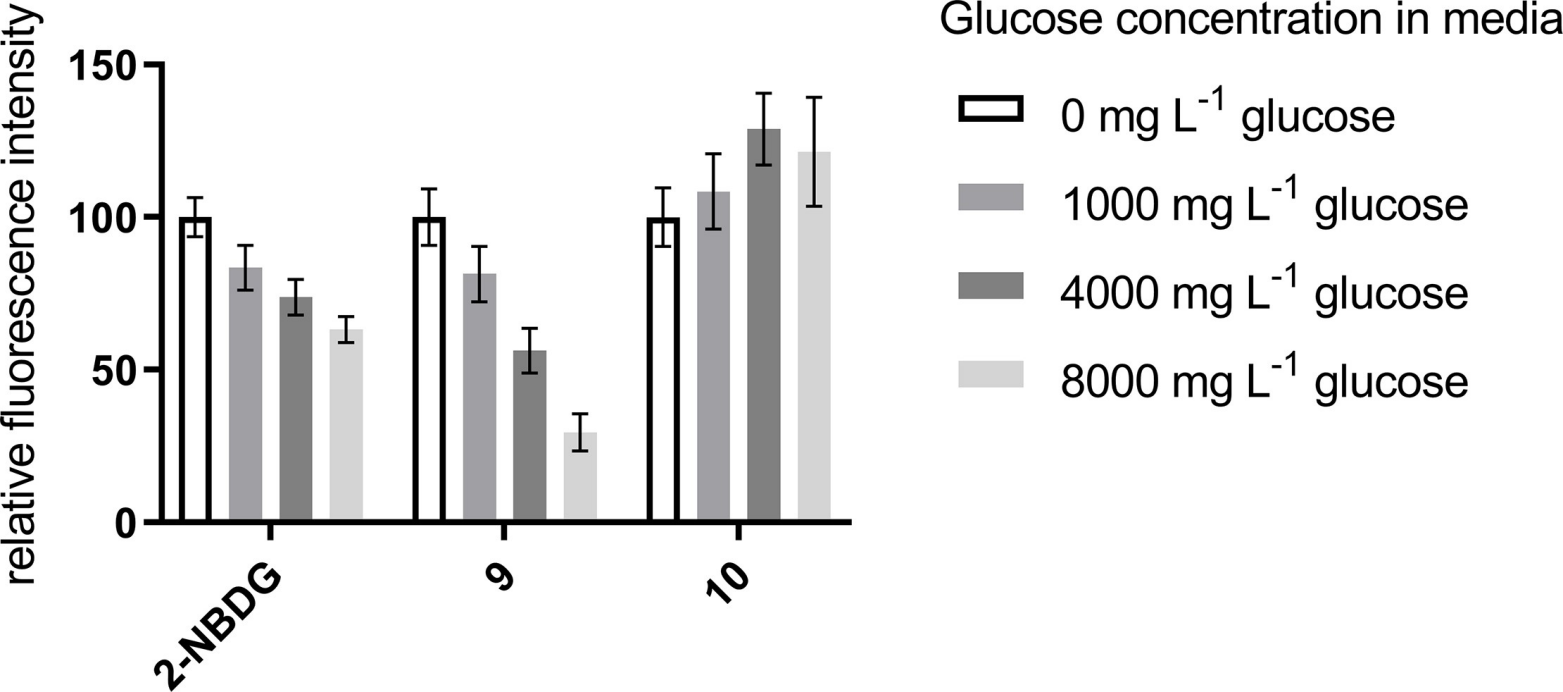
Intracellular fluorescence intensity for DLD-1 colon carcinoma cells dosed with NBD-conjugates at different glucose concentrations.

Warburg effect targeting compounds are expected to compete with glucose for cellular uptake via glucose transporters but measuring the competition directly would require a knowledge of which GLUTs are involved. Instead, we have compared the intracellular fluorescence in the presence of different glucose concentrations to investigate the glucose dependence of the uptake of the NBD-conjugates. Fig 4 shows the relative fluorescence intensity for DLD-1 human colon carcinoma cells in cell culture media containing varying amounts of glucose, and dosed with 2-NBDG, **9** or **10** for 4 h. The range of glucose concentrations used were selected to be similar to those present in glucose-free (0 mg L^-1^), low glucose media (1000 mg L^-1^) and high glucose media (4500 mg L^-1^).

Intracellular fluorescence intensities were measured for a minimum of eight 30x30 μm^2^ regions of DLD-1 colon carcinoma cells grown in monolayer and dosed with NBD-conjugates (50 μM) at increasing glucose concentrations. Examples are shown in S3 Fig. They are shown relative to the fluorescence for cells dosed with each compound in the absence of glucose which have in each case been normalised to 100. Error bars represent standard deviations.

The results reveal that the intracellular fluorescence, and therefore uptake of the compound, decreases with increasing glucose concentration. The cellular uptake of **9** is inhibited by glucose and is so to a greater extent than is the uptake of 2-NBDG. The uptake of **10** is unaffected by glucose concentration, therefore the higher impact of glucose concentration observed for **9** is consistent with uptake via the glucose transporter. An unpaired t-test shows that the differences between the fluorescence levels of 9 and 10 at 4000 mg L^-1^ and 8000 mg L^-1^ are significant at a p < 0.05. This suggests that conjugates of **8** should successfully exploit overexpression of glucose transporters by cancer cells.

Whilst targeting vector **8** has been designed to target GLUT-1, this study does not enable identification of the specific glucose transporters 2-NBDG or **9** are internalised through, and it is possible the greater glucose dependence of the uptake of **9** is due to internalisation by other GLUT isoforms. The greater dependence relative to that of 2-NBDG is consistent with a lower contribution from uptake pathways which are not dependent on glucose concentration such as passive diffusion.

#### Distribution through solid tumour models

**Fig 5:**
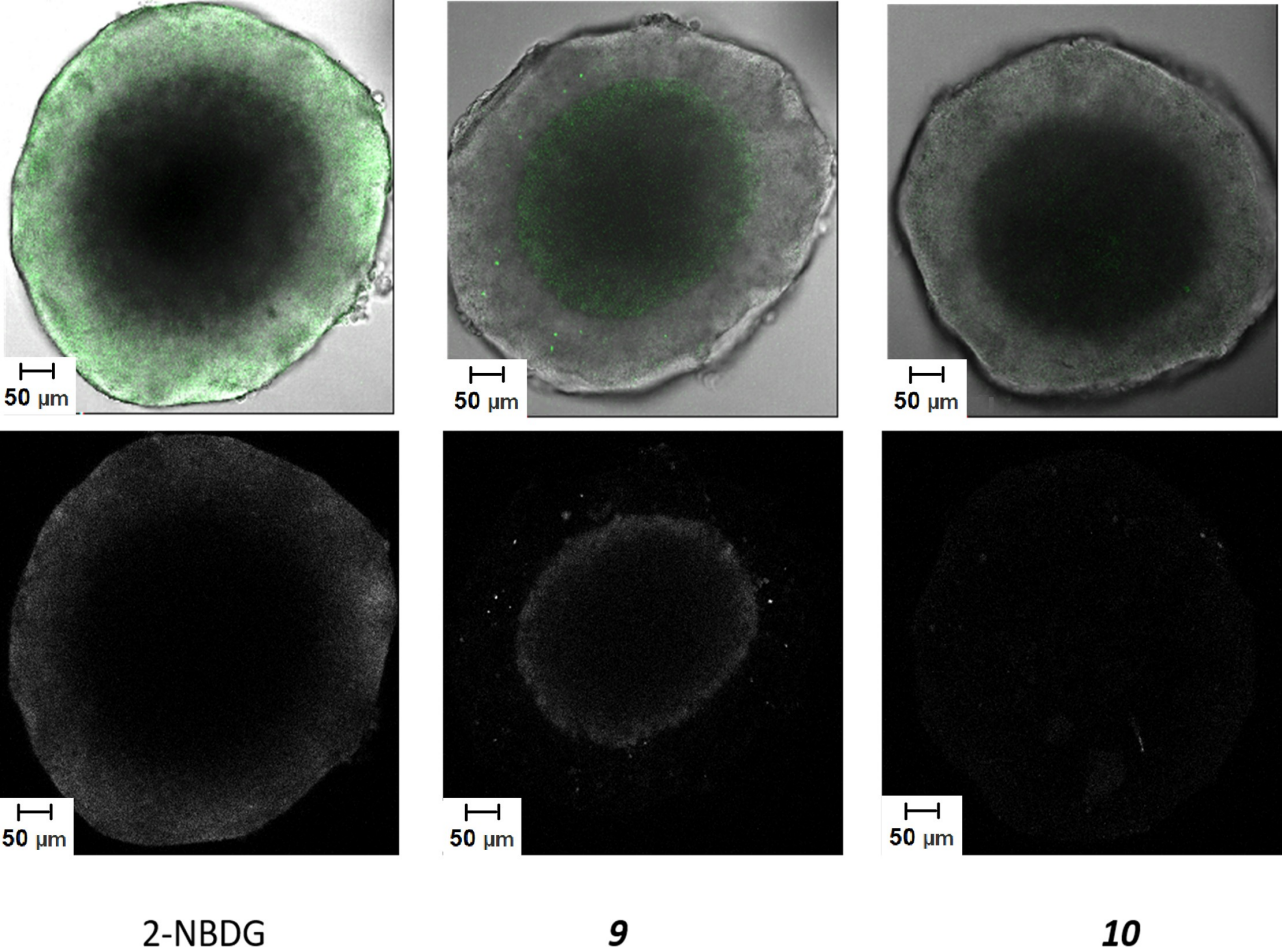
Brightfield and fluorescence images of spheroids treated with 2-NBDG, 9 and 10.

Unlike monolayer models, spheroids contain different regions, resembling those seen in solid tumours, as cells are at different distances from the nutrient source.[54] The cells on the edge of the spheroid are actively proliferating, similar to the cells of a solid tumour in close proximity to blood vessels.[55] Depending on their size, spheroids can develop hypoxic regions, where cells are quiescent, and necrotic cores where most of the cells are dead.[2, 56] These different regions of spheroids are expected to have different glucose requirements, transporter expression and glucose availability that will impact delivery of Warburg effect targeting compounds. The expression of GLUT-1 and GLUT-3 transporters has been demonstrated to be hypoxia-responsive, with a 10-fold increase in GLUT-1 protein expression observed for adipocyte cells cultured in hypoxic compared to normoxic conditions.[57, 58] To explore this, confocal images of the cross sections of spheroids dosed with 2-NBDG, **9** and **10** were examined (Fig 5).

Overlays of brightfield (greyscale) and NBD fluorescence (green) (top row), and confocal images (NBD fluorescence) (bottom row) for cross sections of spheroids which were dosed with 2-NBDG, **9** and **10** at 50 μM for 4 h.

Uptake of **10** is very low, and distribution is even throughout the spheroid cross section, illustrating no selective uptake by cells in different regions. This is consistent with PEG inhibiting cellular uptake, as has previously been reported.[59, 60] 2-NBDG fluorescence, which is limited to the periphery of the spheroid, is expected to mimic glucose uptake by cells throughout a spheroid and therefore be indicative of Warburg effect targeting. Spheroids dosed with **9** however, have a markedly different fluorescence distribution. The uptake of **9** is primarily observed at a distance of approximately 80 μm from the surface of the spheroid, the distance around which hypoxia has been shown to develop,[55] and decreases towards the necrotic core of the spheroid. This observation is consistent with strongly glucose dependent uptake of **9**, as fluorescence is observed in a region with reduced nutrient availability and therefore lower extracellular glucose concentration. As hypoxia is associated with overexpression of some GLUT isoforms, the difference in fluorescence distribution between 2-NBDG and **9** may be due the glucose analogues being internalised by different transporters. It may also result from a higher level of non-selective uptake of 2-NBDG. Consistent with this hypothesis is the observation that the intensity of the fluorescence of cells treated with 2-NBDG is substantially higher than that seen following treatment with **9** (S1 Table) and a conclusion that might be drawn from Fig 4 is that half or more of the uptake of NBDG is not dependent on the glucose concentration.

## Conclusions

A Warburg effect targeting vector (**8**), with the capability to conjugate with many compounds, has been successfully synthesised, conjugated with a fluorophore and evaluated. This targeting vector has demonstrated the ability to bind to HK and thereby inhibit HK catalysed phosphorylation of glucose, an indicator of the desired interaction between the glucose analogue and the enzyme. *In vitro*, the novel Warburg effect targeting vector **8** provides distinct advantages over glucosamine for exploiting the Warburg effect to increase selective uptake by cancer cells. Results indicate cellular uptake of **8** and its fluorescent analogue (**9**) is through glucose transporters that cancer cells overexpress. It is possible that inhibition of the cellular uptake by PEG, while leading to lower levels of accumulation, increases the selectivity because the GLUT dependent pathways become more dominant. Additionally, this vector may provide the potential to target the difficult to treat hypoxic regions of tumours when conjugated to anticancer agents.

## Supporting information

S1 Fig Plot of the relative rate of HK catalysed glucose phosphorylation in the presence of increasing concentration of compounds, up to 714 μM. (PNG)

S2 Fig Fluorescence emission spectra of 2-NBDG, *9* and *10* (50 μM) in H2O. Emission scans were collected between 510 and 700 nm using an excitation wavelength of 488 nm.
(PNG)

S3 Fig **Confocal images of NBD fluorescence (green), brightfield (greyscale) and overlay of images (from left to right) for monolayer DLD-1 cells in glucose-free media, dosed with 2-NBDG, *9* and *10* (50** μ**M for 2 h)**
(PNG)

S1 Table Raw data values of fluorescence intensities for 3 regions of DLD-1 cells in glucose-free media dosed with each compound (50 μM) for 2 h. Fluorescence intensities were determined by quantification with LAS AF Lite.
(DOCX)

S1 Dataset Minimal underlying data set. (DOCX)

## Abbreviations

2-NBDG: 2-(N-(7-nitrobenz-2-oxa-1,3-diazol-4-yl)amino)-2-deoxy-D-glucose
ATP: adenosine triphosphate
G6P: glucose-6-phosphate
GLUT: facilitative-diffusion glucose transporter
NAD: nicotinamide adenine dinucleotide

## References

[1] Chen Y, Hu L. Design of anticancer prodrugs for reductive activation. Med Res Rev. 2009;29(1):29–64. 10.1002/med.20137 . 18688784

[2] Minchinton AI, Tannock IF. Drug penetration in solid tumours. Nat Rev Cancer. 2006;6(8):583–92. 10.1038/nrc1893 . 16862189

[3] Srinivasarao M, Low PS. Ligand-Targeted Drug Delivery. Chemical reviews. 2017;117(19):12133–64. 10.1021/acs.chemrev.7b00013 . 28898067

[4] Elf SE, Chen J. Targeting glucose metabolism in patients with cancer. Cancer. 2014;120(6):774–80. 10.1002/cncr.28501 . 24374503 PMC4507501

[5] Zhao Y, Butler EB, Tan M. Targeting cellular metabolism to improve cancer therapeutics. Cell Death and Disease. 2013;4(e532). 10.1038/cddis.2013.60 23470539 PMC3613838

[6] Cairns RA, Harris IS, Mak TW. Regulation of cancer cell metabolism. Nat Rev Cancer. 2011;11(2):85–95. 10.1038/nrc2981 . 21258394

[7] Allen TM. Ligand-targeted therapeutics in anticancer therapy. Nat Rev Cancer. 2002;2(10):750–63. 10.1038/nrc903 . 12360278

[8] Reddy LH. Drug delivery to tumours: recent strategies. The Journal of pharmacy and pharmacology. 2005;57(10):1231–42. 10.1211/jpp.57.10.0001 . 16259751

[9] Chen S, Zhao X, Chen J, Chen J, Kuznetsova L, Wong SS, et al Mechanism-based tumor-targeting drug delivery system. Validation of efficient vitamin receptor-mediated endocytosis and drug release. Bioconjug Chem. 2010;21:979–87. 10.1021/bc9005656 20429547 PMC3036843

[10] Warburg O. The metabolism of carcinoma cells. The Journal of Cancer Research. 1925;9(1):148–63. 10.1158/jcr.1925.148

[11] Hanahan D, Weinberg RA. Hallmarks of cancer: the next generation. Cell. 2011;144(5):646–74. 10.1016/j.cell.2011.02.013 . 21376230

[12] Wahl RL. Targeting glucose transporters for tumor imaging: "sweet" idea, "sour" result. J Nucl Med. 1996;37:1038–41. 8683297

[13] Scheepers A, Joost H, Schurmann A. The glucose transporter families SGLT and GLUT: molecular basis of normal and aberrant function. J Parenter Enteral Nutr. 2004;28(5):364–71. 10.1177/0148607104028005364 15449578

[14] Granchi C, Minutolo F. Anti-cancer agents that counteract tumor glycolysis. Chem Med Chem. 2012;7(8):1318–50. 10.1002/cmdc.201200176 22684868 PMC3516916

[15] Weissleder R. Molecular imaging in cancer. Science. 2006;312(5777):1168–71. 10.1126/science.1125949 . 16728630

[16] Calvaresi EC, Hergenrother PJ. Glucose conjugation for the specific targeting and treatment of cancer. Chem Sci. 2013;4:2319–33. 10.1039/C3SC22205E 24077675 PMC3784344

[17] Pettenuzzo A, Pigot R, Ronconi L. Metal-based glycoconjugates and their potential in targeted anticancer chemotherapy. Metallodrugs. 2015;1:36–61. 10.1515/medr-2015-0002

[18] Ma J, Wang Q, Huang Z, Yang X, Nie Q, Hao W, et al Glycosylated Platinum(IV) Complexes as Substrates for Glucose Transporters (GLUTs) and Organic Cation Transporters (OCTs) Exhibited Cancer Targeting and Human Serum Albumin Binding Properties for Drug Delivery. Journal of medicinal chemistry. 2017;60(13):5736–48. 10.1021/acs.jmedchem.7b00433 . 28603992

[19] Patra M, Johnstone TC, Suntharalingam K, Lippard SJ. A potent glucose-platinum conjugate exploits glucose transporters and preferentially accumulates in cancer cells. Angewandte Chemie. 2016;55(7):2550–4. 10.1002/anie.201510551 . 26749149 PMC4752825

[20] Bowen ML, Lim NC, Ewart CB, Misri R, Ferreira CL, Hafeli U, et al Glucosamine conjugates bearing *N*,*N*,*O*-donors: potential imaging agents utilizing the [M(CO)_3_]^+^ core (M = Re, Tc). Dalton Trans. 2009;(42):9216–27. 10.1039/b914310f . 20449199

[21] Gould GW, Thomas HM, Jess TJ, Bell GI. Expression of human glucose transporters in *Xenopus* oocytes: kinetic characterization and substrate specificities of the erythrocyte, liver, and brain isoforms. Biochem. 1991;30:5139–45. 2036379 10.1021/bi00235a004

[22] Medina RA, Owen GI. Glucose transporters: expression, regulation and cancer. Biological Res. 2002;35:9–26. 10.4067/s0716-97602002000100004 12125211

[23] O'Neil RG, Wu L, Mullani N. Uptake of a fluorescent deoxyglucose analog (2-NBDG) in tumor cells. Mol Imaging Biol. 2005;7(6):388–92. 10.1007/s11307-005-0011-6 . 16284704

[24] Patra M, Awuah SG, Lippard SJ. Chemical approach to positional isomers of glucose-platinum conjugates reveals specific cancer targeting through glucose-transporter-mediated uptake *in vitro* and *in vivo*. Journal of the American Chemical Society. 2016;138(38):12541–51. 10.1021/jacs.6b06937 . 27570149 PMC5042873

[25] Cao J, Cui S, Li S, Du C, Tian J, Wan S, et al Targeted cancer therapy with a 2-deoxyglucose-based adriamycin complex. Cancer Res. 2013;73(4):1362–73. 10.1158/0008-5472.CAN-12-2072 . 23396585

[26] Schibli R, Dumas C, Petrig J, Spadola L, Scapozza L, Garcia-Garayoa E, et al Synthesis and in vitro characterisation of organometallic rhenium and technetium glucose complexes against Glut 1 and hexokinase. Bioconjug Chem. 2005;16:105–12. 10.1021/bc049774l 15656581

[27] Bowen ML, Chen ZF, Roos AM, Misri R, Hafeli U, Adam MJ, et al Long-chain rhenium and technetium glucosamine conjugates. Dalton Trans. 2009;(42):9228–36. 10.1039/b914309b . 20449200

[28] Legeay JC, Eynde JJV, Bazureau JP. Sequential synthesis of a new analogue of amlodipine bearing a short amino polyethyleneglycol chain. Tetrahedron. 2007;63(48):12081–6. 10.1016/j.tet.2007.08.111

[29] Liu L, Dietsch H, Schurtenberger P, Yan M. Photoinitiated coupling of unmodified monosaccharides to iron oxide nanoparticles for sensing proteins and bacteria. Bioconjug Chem. 2009;20:1349–55. 10.1021/bc900110x 19534519 PMC2733941

[30] Sakamoto J, Takita C, Koyama T, Hatano K, Terunuma D, Matsuoka K. Use of a recycle-type SEC method as a powerful tool for purification of thiosialoside derivatives. Carbohydrate research. 2008;343(16):2735–9. 10.1016/j.carres.2008.05.014 . 18550037

[31] He Q, Wang Z, Chen X, Jiang ZH, Ai W, Jiang D. An efficient way to the synthesis of 2-deoxy-2-[(7-nitro-2,1,3-benzoxadiazol-4-yl)amino]-*b*-D-glucopyranose (2-NBDG) via 7-nitro-2,1,3-benzoxadiazol-4-ly chloride (NBD-Cl). Letters in Org Chem. 2013;10:538–40.

[32] Yadav JS, Satyanarayana M, Raghavendra S, Balanarsaiah E. Chemoselective hydrolysis of terminal isopropylidene acetals in acetonitrile using molecular iodine as a mild and efficient catalyst. Tetrahedron Lett. 2005;46(50):8745–8. 10.1016/j.tetlet.2005.10.043

[33] Monrad RN, Madsen R. Rhodium-catalysed decarbonylation of aldoses. J Org Chem. 2007;72:9782–5. 10.1021/jo7017729 17979290

[34] Huber G, Rossi A. Uber tribenzyl-D-glucofuranoside, eine neue gruppe von heilmitteln auf dem kohlenhydratgebiet. Helv Chim Acta. 1968;51(6):1195–202. 10.1002/hlca.19680510602 5697769

[35] Du Y, Kong F. Synthesis and glycosidic reaction of 1,2-anhydromanno-, lyxo-, gluco-, and xylofuranose perbenzyl ethers. J Carbohydr Chem. 1996;15(7):797–817. 10.1080/07328309608005693

[36] Lee DS, Perlin AS. Acid-catalyzed conversion of 2-*O*-(2-hydroxypropyl)-D-glucose derivatives into 1,2-*O*-(1-methyl-1,2-ethanediyl)-D-glucose acetals. Studies related to *O*-(2-hydroxypropyl)cellulose. Carbohydrate research. 1984;125:265–82.

[37] Ghorai S, Mukhopadhyay R, Kundu AP, Bhattacharjya A. Intramolecular 1,3-dipolar nitrone and nitrile oxide cycloaddition of 2- and 4-*O*-allyl and propargyl glucose derivatives: a versatile approach to chiral cyclic ether fused isoxazolidines, isoxazolines and isoxazoles. Tetrahedron. 2005;61(12):2999–3012. 10.1016/j.tet.2005.01.119

[38] Surfraz MB-U, Akhtar M, Allemann RK. Bis-benzyl protected 6-amino cyclitols are poisonous to Pd/C catalysed hydrogenolysis of benzyl ethers. Tetrahedron Lett. 2004;45(6):1223–6. 10.1016/j.tetlet.2003.11.130

[39] Sajiki H, Kuno H, Hirota K. Suppression effect of the Pd/C-catalyzed hydrogenolysis of a phenolic benzyl protective group by the addition of nitrogen-containing bases. Tetrahedron Lett. 1998;39:7127–30.

[40] Sajiki H. Selective inhibition of benzyl ether hydrogenolysis with Pd/C due to presence of ammonia, pyridine or ammonium acetate. Tetrahedron Lett. 1995;36(20):3465–8.

[41] Wuts PGM, Greene TW. Greene's Protective Groups in Organic Synthesis: Chapter 7. 5th ed: Wiley; 2014.

[42] Jarowicki K, Kocienski P. Protecting groups. J Chem Soc, Perkin Trans 1. 2001;(18):2109–35. 10.1039/b103282h

[43] Matwiejuk M, Thiem J. New method for regioselective glycosylation employing saccharide oxyanions. Eur J Org Chem. 2011;2011(29):5860–78. 10.1002/ejoc.201100861

[44] Molteni C, Parrinello M. Glucose in aqueous solution by first principles of molecular dynamics. Journal of the American Chemical Society. 1998;120:2168–71.

[45] Brady JW. Molecular dynamics simulations of α-D-glucose in aqueous solution. Journal of the American Chemical Society. 1989;111:5155–65.

[46] Gurst JE. NMR and the structure of D-glucose. J Chem Ed. 1991;68(12):1003–4.

[47] Curatolo W, Neuringer LJ, Ruben D, Haberkorn R. Two-dimensional *J*-resolved ^1^H-nuclear magnetic resonance spectroscopy of α,β-D-glucose at 500 MHz. Carbohydrate research. 1983;112:297–300.

[48] Ferreira CL, Ewart CB, Bayly SR, Patrick BO, Steele J, Adam MJ, et al Glucosamine conjugates of tricarbonylcyclopentadienyl rhenium(I) and technetium(I) cores. Inorg Chem. 2006;45:6979–87. 10.1021/ic0605672 16903757

[49] Bertoni JM. Competitive inhibition of rat brain hexokinase by 2-deoxyglucose, glucosamine, and metrizamide. J Neurochem. 1981;37(6):1523–8. 7334375 10.1111/j.1471-4159.1981.tb06322.x

[50] Hofmann M, Roitsch T. The hexokinase inhibitor glucosamine exerts a concentration dependent dual effect on protein kinase activity *in vitro*. J Plant Physiol. 2000;157:13–6. 10.1016/s0176-1617(00)80129-7

[51] Cai H, Peng F. 2-NBDG fluorescence imaging of hypermetabolic circulating tumor cells in mouse xenograft model of breast cancer. J Fluoresc. 2013;23(1):213–20. 10.1007/s10895-012-1136-z . 23054302 PMC4592774

[52] Chitcholtan K, Sykes PH, Evans JJ. The resistance of intracellular mediators to doxorubicin and cisplatin are distinct in 3D and 2D endometrial cancer. J Transl Med. 2012;10(38):1–16. 10.1186/1479-5876-10-38 22394685 PMC3316127

[53] Luo Z, Tikekar RV, Samadzadeh KM, Nitin N. Optical molecular imaging approach for rapid assessment of response of individual cancer cells to chemotherapy. J Biomed Opt. 2012;17(10):106006/1-/8. 10.1117/1.JBO.17.10.106006 23224005 PMC3461756

[54] Elliott NT, Yuan F. A review of three-dimensional *in vitro* tissue models for drug discovery and transport studies. J Pharm Sci. 2011;100(1):59–74. 10.1002/jps.22257 . 20533556

[55] Kim BJ, Hambley TW, Bryce NS. Visualising the hypoxia selectivity of cobalt(III) prodrugs. Chem Sci. 2011;2(11):2135–42. 10.1039/c1sc00337b

[56] Bryce NS, Zhang JZ, Whan RM, Yamamoto N, Hambley TW. Accumulation of an anthraquinone and its platinum complexes in cancer cell spheroids: the effect of charge on drug distribution in solid tumour models. Chem Commun. 2009;(19):2673–5. 10.1039/b902415h . 19532917

[57] Wood IS, Wang B, Lorente-Cebrian S, Trayhurn P. Hypoxia increases expression of selective facilitative glucose transporters (GLUT) and 2-deoxy-D-glucose uptake in human adipocytes. Biochemical and biophysical research communications. 2007;361(2):468–73. 10.1016/j.bbrc.2007.07.032 17658463 PMC2211375

[58] Airley RE, Mobasheri A. Hypoxic regulation of glucose transport, anaerobic metabolism and angiogenesis in cancer: novel pathways and targets for anticancer therapeutics. Chemotherapy. 2007;53(4):233–56. 10.1159/000104457 . 17595539

[59] Hatakeyama H, Akita H, Harashima H. The polyethyleneglycol dilema: Advantages and disadvantages of PEGylation of liposomes for systemic genes and nucleic acids delivery to tumors. Biol Pharm Bull. 2013;36(6):892–9. 23727912 10.1248/bpb.b13-00059

[60] Fang Y, Xue J, Gao S, Lu A, Yang D, Jiang H, et al Cleavable PEGylation: a strategy for overcoming the "PEG dilemma" in efficient drug delivery. Drug Deliv. 2017;24(sup1):22–32. 10.1080/10717544.2017.1388451 . 29069920 PMC8812578

