# Supplementary figures and images for: A Warburg effect targeting vector designed to increase the uptake of compounds by cancer cells demonstrates glucose and hypoxia dependent uptake
Source: PLoS One. 2019 Jul 15;14(7):e0217712. doi: 10.1371/journal.pone.0217712 (PMC6629077; doi:10.1371/journal.pone.0217712)

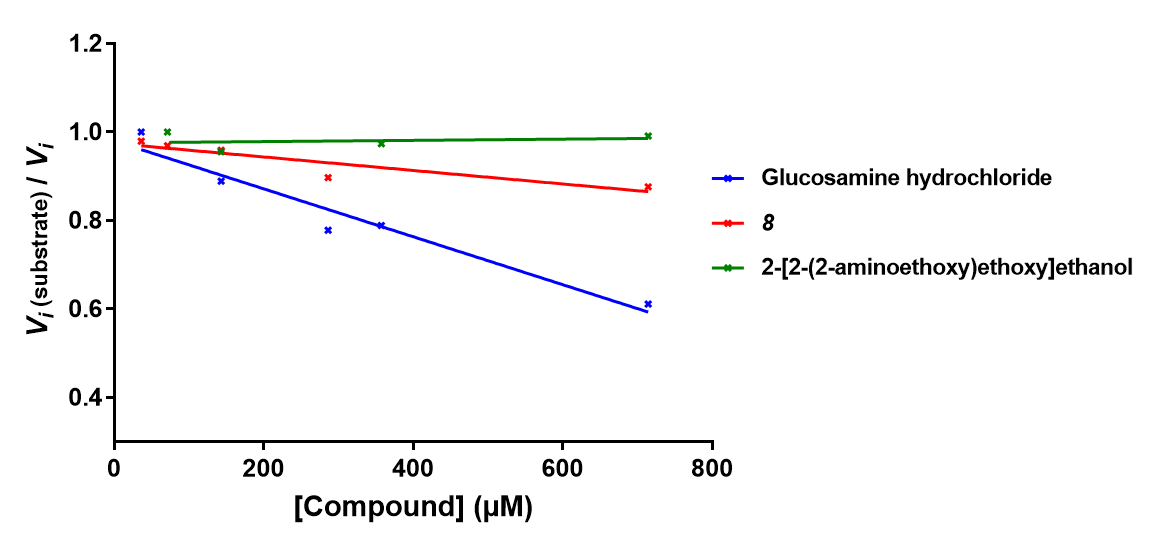

Supplement: S1 Fig — (PNG) [file pone.0217712.s001.png]

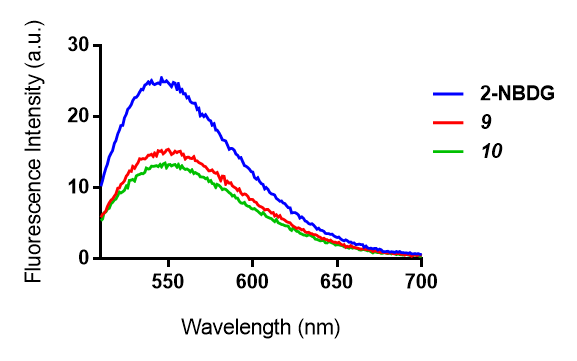

Supplement: S2 Fig — Emission scans were collected between 510 and 700 nm using an excitation wavelength of 488 nm. (PNG) [file pone.0217712.s002.png]

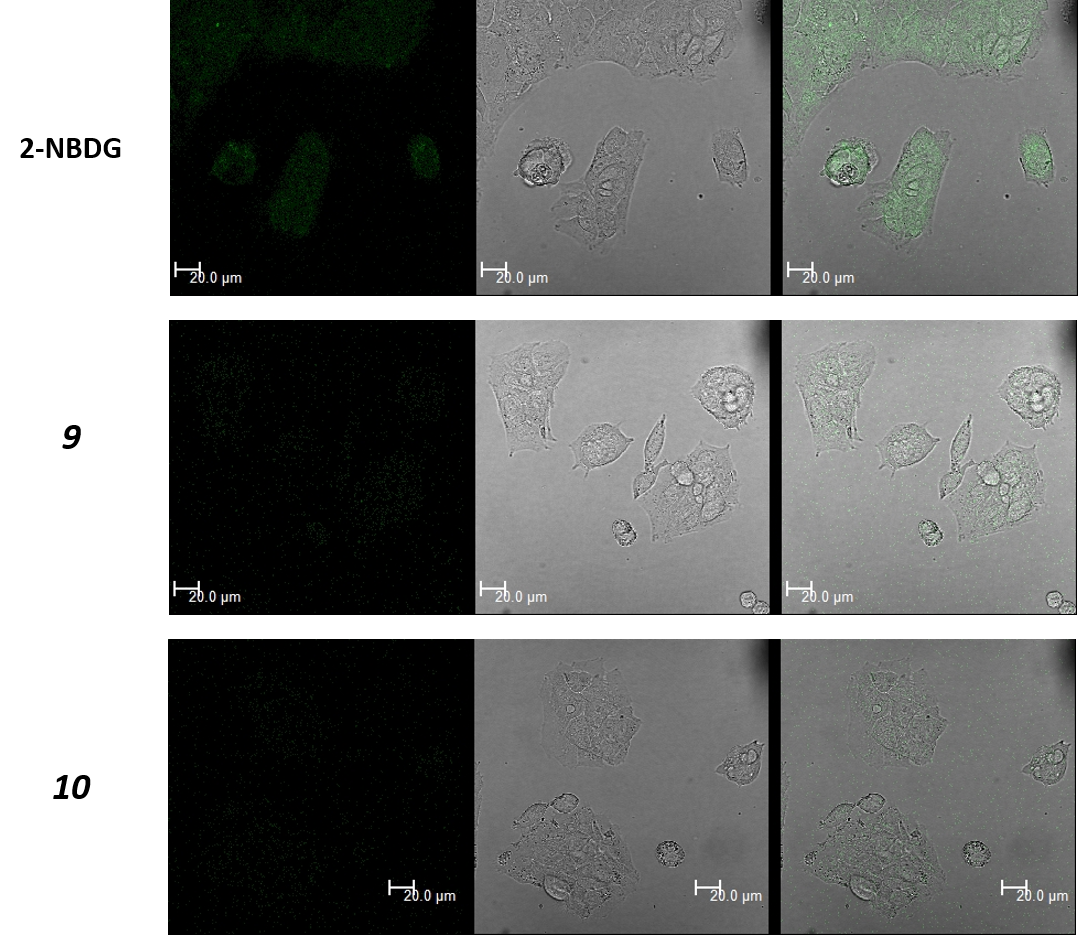

Supplement: S3 Fig — Confocal images of NBD fluorescence (green), brightfield (greyscale) and overlay of images (from left to right) for monolayer DLD-1 cells in glucose-free media, dosed with 2-NBDG, 9 and 10 (50 μM for 2 h) (PNG) [file pone.0217712.s003.png]
