# Supplementary material for: A Warburg effect targeting vector designed to increase the uptake of compounds by cancer cells demonstrates glucose and hypoxia dependent uptake
Source: PLoS One. 2019 Jul 15;14(7):e0217712. doi: 10.1371/journal.pone.0217712 (PMC6629077; doi:10.1371/journal.pone.0217712)
Supplement: S1 Table — Fluorescence intensities were determined by quantification with LAS AF Lite. (DOCX) [file pone.0217712.s004.docx]

A Warburg effect targeting vector designed to increase the uptake of compounds by cancer cells demonstrates glucose and hypoxia dependent uptake

Alexandra Glenister,^§^ Michela I. Simone,^†^ and Trevor W. Hambley^§^

^§^ School of Chemistry, University of Sydney, New South Wales, 2006, Australia

^†^ Discipline of Chemistry, Priority Research Centre for Chemical Biology & Clinical Pharmacology, University of Newcastle, Callaghan, New South Wales, 2308, Australia.

# Supporting information

Fluorescence spectrometry, plot of initial rate of glucose phosphorylation from hexokinase inhibition assay, cell images for Fig 4, and raw data for Fig 4, S1 Fig, and S1 Table.

# Glucose Dependent Uptake in Monolayer Cell Culture

Sample data for the fluorescence intensity of cell regions (30 µm x 30 µm) of confocal images of monolayer DLD-1 cells, in media containing 0 mg L^-1^ glucose, dosed with compounds are shown in Table S1. For plots comparing fluorescence intensity (Fig 4), the average of a minimum of 8 cell regions (30 µm x 30 µm) was calculated and the results presented as a percentage of fluorescence intensity compared to cells dosed with the compound at 0 mg L^-1^ glucose.

Table S1. Raw data values of fluorescence intensities for 3 regions of DLD-1 cells in glucose-free media dosed with each compounds (50 µM) for 2 h. Fluorescence intensities were determined by quantification with LAS AF Lite.

| Compound | Fluorescence Intensity (A. U.) of cell region | | | | | | | | | |
| --- | --- | --- | --- | --- | --- | --- | --- | --- | --- | --- |
|  |  |  |  |  | |  |  | |  |  |
| 2-NBDG | 2729 | | | | 2771 | | | 2238 | | |
| 9 | 402 | | | | 430 | | | 465 | | |
| 10 | 362 | | | | 390 | | | 406 | | |

# Synthesis

2-[2-(2-azidoethoxy)ethoxy]ethanol was synthesised by a modification of the method of Legeay *et al*.[[28](#_ENREF_28)] 2-[2-(2-aminoethoxy)ethoxy]ethanol was synthesised by the procedure of Liu *et al*.[[29](#_ENREF_29)] 2-[2-(2-azidoethoxy)ethoxy]ethyl mesylate was synthesised by a modification of the procedure of Sakamoto *et al*.[[30](#_ENREF_30)] 2-NBDG was synthesised by the method of He *et al*.,[[31](#_ENREF_31)] and purified on a Sephadex LH-20 column eluting with H_2_O.

## 1,2-O-isopropylidene-α-D-glucofuranose (1)

1,2*-O-*isopropylidene-α-D-glucofuranose was prepared by a modification of the method of Yadav *et al*.[[32](#_ENREF_32)] Iodine (1.47 g, 5.8 mmol, 0.3 eq.) was added to a solution of 1,2:5,6-di-*O*-isopropylidene-α-D-glucofuranose (5.00 g, 19.2 mmol) in MeCN (270 mL). H_2_O (2 mL) was added, and the reaction mixture was stirred at room temperature for 7 h. The reaction was quenched with saturated aqueous Na_2_S_2_O_3_ solution and extracted into EtOAc (5 x 200 mL). The organic layers were dried over anhydrous Na_2_SO_4_, the solvent removed and the crude mixture was purified by column chromatography (silica) using EtOAc/hexane 2:1, EtOAc and EtOAc/MeOH 1:0.01 to give the product as a white powder (3.29 g, 14.9 mmol, 78% yield). Rf 0.23 (EtOAc, silica); ${[\alpha]}_{D}^{26}$ -18.3˚ (*c* 1.00, H_2_O) (lit. ${[\alpha]}_{D}^{22}$ -12.0˚ (*c* 1.0, H_2_O)[[33](#_ENREF_33)]); ^1^H NMR (400 MHz, MeOD): δ 5.86 (d, *J* 3.6 Hz, 1H, CH), 4.47 (d, *J* 3.6 Hz, 1H, CH), 4.20 (d, *J* 2.8 Hz, 1H, CH), 4.01 (dd, *J* 8.4, 2.8 Hz, 1H, CH), 3.89 (ddd, *J* 8.4, 6.0, 3.2 Hz, 1H, CH), 3.75 (dd, *J* 11.6, 3.2 Hz, 1H, CH (glucose C6)), 3.59 (dd, *J* 11.6, 6.0 Hz, 1H, CH (glucose C6)), 1.45 (s, 3H, CH_3_), 1.24 (s, 3H, CH_3_); ^13^C NMR (100 MHz, MeOD): δ 112.7, 106.4, 86.5, 81.4, 75.5, 70.4, 65.3, 27.0, 26.4.

## 3,5,6-tri-O-benzyl-1,2-O-isopropylidene-α-D-glucofuranose (2)

Under N_2_, **1** (1.00 g, 4.5 mmol) was dissolved in anhydrous DMF (24 mL). Sodium hydride in a 60% oily dispersion (0.90 g, 22.5 mmol, 5 eq.) was added portionwise with vigorous stirring. After stirring for 30 min, benzyl bromide (2.80 mL, 22.5 mmol, 5 eq.) was added dropwise. The reaction mixture was stirred at room temperature, under N_2_ for 24 h and then treated with H_2_O (150 mL) and neutralised with 1 M hydrochloric acid. The product was extracted into DCM (3 x 150 mL), and the organic layer was dried over Na_2_SO_4_ and the solvent removed. The product was purified by column chromatography (silica) with hexane/EtOAc 8:1 eluent to yield **2** as a light yellow oil (2.01 g, 4.1 mmol, 90% yield).

Rf 0.46 (hexane/EtOAc 2:1, silica); ${[\alpha]}_{D}^{26}$ -33.8˚ (*c* 2.03, CHCl_3_) (lit. ${[\alpha]}_{D}^{20}$ -36˚ (*c* 1.0, CHCl_3_)[[34](#_ENREF_34)]; ${[\alpha]}_{D}^{25}$ -33˚ (*c* 9.3, CHCl_3_)[[35](#_ENREF_35)]); ^1^H NMR (400 MHz, MeOD): δ 7.34-7.19 (m, 15H, 15 x aromatic CH), 5.85 (d, *J* 3.6 Hz, 1H, CH), 4.70-4.62 (m, 3H, CH and CH_2_), 4.51 (s, 2H, CH_2_), 4.42 (dd, *J* 11.6, 3.6 Hz, 2H, CH_2_), 4.29 (dd, *J* 9.2, 2.8 Hz, 1H, CH), 4.03 (d, *J* 2.8 Hz, 1H, CH), 3.95 (ddd, *J* 9.2, 4.8, 2.0 Hz, 1H, CH), 3.86 (dd, *J* 10.8, 2.0 Hz, 1H, CH (glucose C6)), 3.61 (dd, *J* 10.8, 4.8 Hz, 1H, CH (glucose C6)), 1.44 (s, 3H, CH_3_), 1.29 (s, 3H, CH_3_); ^13^C NMR (100 MHz, MeOD): δ 139.9, 139.7, 139.1, 129.4, 129.3, 129.3, 128.9, 128.8, 128.7, 128.6, 128.6, 128.5, 112.9, 106.4, 83.0, 82.9, 80.1, 76.9, 74.3, 73.3, 72.9, 71.2, 27.1, 26.5.

## Methyl 3,5,6-tri-O-benzyl-α-D-glucofuranoside (3a) and methyl 3,5,6-tri-O-benzyl-β-D-glucofuranoside (3b)

The method of Lee and Perlin[[36](#_ENREF_36)] was used for the synthesis of methyl 3,5,6-tri*-O-*benzyl-α-D-glucofuranoside and methyl 3,5,6-tri*-O-*benzyl-β-D-glucofuranoside. **2** (1.00 g, 2.04 mmol) was dissolved in MeOH (20 mL) under N_2_. Amberlite IR-120 (H^+^) ion exchange resin (10.25 g) was added, and the reaction was refluxed under N_2_ for 24 h. The reaction mixture was filtered, the filtrate evaporated and the crude mixture separated by column chromatography (silica) with hexane/EtOAc 4:1 and hexane/EtOAc 2:1 to yield *3a* and *3b* as colourless oils (**3a**: 0.34 g, 0.73 mmol, 36% yield; **3b**: 0.31 g, 0.67 mmol, 33% yield).

##### 3a

Rf 0.53 (hexane/EtOAc 2:1, silica); ${[\alpha]}_{D}^{26}$ +21.7˚ (*c* 0.40, CHCl_3_) (lit. ${[\alpha]}_{D}^{28}$ +28.9˚ (*c* 0.32, CHCl_3_)[[37](#_ENREF_37)]); ^1^H NMR (400 MHz, MeOD): δ 7.32-7.23 (m, 15H, 15 x aromatic CH), 4.92 (d, *J* 4.4 Hz, 1H, CH), 4.71 (dd, *J* 11.6, 7.6 Hz, 2H, CH_2_), 4.52 (d, *J* 2.0 Hz, 2H, CH_2_), 4.49 (dd, *J* 11.6, 2.0 Hz, 2H, CH_2_), 4.31 (dd, *J* 7.6, 5.2 Hz, 1H, CH), 4.21 (dd, *J* 4.4, 3.2 Hz, 1H, CH), 4.04 (dd, *J* 5.2, 3.2 Hz, 1H, CH), 3.96 (ddd, *J* 7.6, 5.6, 2.0 Hz, 1H, CH), 3.85 (dd, *J* 10.8, 2.0 Hz, 1H, CH (glucose C6)), 3.66 (dd, *J* 10.8, 5.6 Hz, 1H, CH (glucose C6)), 3.42 (s, 3H, CH_3_); ^13^C NMR (100 MHz, MeOD): δ 140.1, 139.8, 139.5, 129.4, 129.3, 129.2, 128.9, 128.8, 128.8, 128.7, 128.6, 128.5, 104.1, 85.0, 78.3, 77.8, 77.6, 74.3, 73.4, 73.0, 71.7, 56.0.

##### 3b

Rf 0.32 (hexane/EtOAc 2:1, silica); ${[\alpha]}_{D}^{26}$ -58.7˚ (*c* 0.21, CHCl_3_) (lit. ${[\alpha]}_{D}^{28}$ -54.9˚ (*c* 0.25, CHCl_3_)); ^1^H NMR (400 MHz, MeOD): δ 7.35-7.21 (m, 15H, 15 x aromatic CH), 4.78 (s, 1H, CH), 4.66 (dd, *J* 13.6, 11.6 Hz, 2H, CH_2_), 4.54 (s, 2H, CH_2_), 4.47 (d, *J* 4.4 Hz, 2H, CH_2_), 4.44 (d, *J* 4.8 Hz, 1H, CH), 4.35 (dd, *J* 8.8, 4.8 Hz, 1H, CH), 4.18 (s, 1H, CH), 4.01 (ddd, *J* 8.8, 5.2, 2.0 Hz, 1H, CH), 3.94 (dd, *J* 4.4, 0.4 Hz, 1H, CH), 3.91 (dd, *J* 10.8, 2.0 Hz, 1H, CH (glucose C6)), 3.67 (dd, *J* 10.8, 5.2 Hz, 1H, CH (glucose C6)), 3.30 (s, 3H, CH_3_); ^13^C NMR (100 MHz, MeOD): δ 140.1, 139.7, 139.4, 129.3, 129.3, 129.2, 128.9, 128.8, 128.7, 128.7, 128.6, 128.5, 111.5, 84.4, 81.6, 78.6, 77.9, 74.3, 73.3, 72.9, 71.7, 55.9.

## Methyl 2-(2-[2-(2-azidoethoxy)ethoxy]ethoxy)-3,5,6-tri-O-benzyl-α-D-glucofuranoside (4a) and methyl 2-(2-[2-(2-azidoethoxy)ethoxy]ethoxy)-3,5,6-tri-O-benzyl-β-D-glucofuranoside (4b)

**3a** or **3b** (1.55 g, 3.34 mmol) was dissolved in anhydrous DMF (20 mL) under N_2_. Sodium hydride in a 60% oily dispersion (0.31 g, 7.68 mmol, 2.3 eq.) was added portionwise, and the reaction mixture stirred at room temperature for 20 min. 2-[2-(2-azidoethoxy)ethoxy]ethyl methanesulfonate (1.31 g, 5.18 mmol, 1.55 eq.) in anhydrous DMF (1 mL) was added dropwise to the reaction mixture, which was subsequently stirred at 60 ˚C under N_2_ for 1 week. MeOH (20 mL) was added and the mixture stirred for 30 min, before the solvent was removed. The resulting solid was dissolved in EtOAc (60 mL), washed with H_2_O (2 x 50 mL) and dried over Na_2_SO_4_.

##### 4a

The crude mixture was purified by column chromatography (silica) with hexane/EtOAc 4:1 and EtOAc to give the product as a yellow oil (1.61 g, 2.59 mmol, 78% yield). Rf 0.67 (hexane/EtOAc 1:1, silica); ${[\alpha]}_{D}^{26}$ +33.3˚ (*c* 0.25, CHCl_3_); IR (4000–400 cm^-1^): 3064 w, 3031 m (aromatic C-H), 2867 s with left shoulder (alkyl C-H), 2100 s (N_3_), 1606 w, 1497 and 1454 s (aromatic C=C), 1355 s, 1304 m, 1193 m, 1175 w, 1100 s (C-O), 1058 s (C-O), 1027 s (C-O), 914 m, 850 w, 807 w, 735 and 697 s (aromatic C-H), 606 w, 527 w, 460 w; LRMS (ESI+): m/z calculated 644.29 ([M+Na]^+^), found 644.13 ([M+Na]^+^); ^1^H NMR (400 MHz, MeOD): δ 7.31-7.24 (m, 15H, 15 x aromatic CH), 5.01 (d, *J* 4.4 Hz, 1H, CH), 4.70 (d, *J* 12.0 Hz, 2H, CH_2_), 4.51 (dt, *J* 1.6, 5.6 Hz, 4H, 2 x CH_2_), 4.28 (dd, *J* 6.8, 6.0 Hz, 1H, CH), 4.15 (dd, *J* 6.0, 4.0 Hz, 1H, CH), 4.07 (t, *J* 4.0 Hz, 1H, CH), 3.97 (ddd, *J* 10.8, 5.6, 2.0 Hz, 1H, CH (glucose C6)), 3.85 (dd, *J* 10.8, 2.0 Hz, 1H, CH (glucose C6)), 3.74-3.59 (m, 12H, 6 x CH_2_ (PEG)), 3.39 (s, 3H, CH_3_), 3.29 (app. t, *J* 4.8 Hz, 1H, CH); ^13^C NMR (100 MHz, MeOD): δ 140.1, 139.9, 139.6, 129.4, 129.3, 129.2, 128.9, 128.8, 128.7, 128.7, 128.5, 128.5, 103.0, 86.2, 83.2, 78.0, 77.9, 74.3, 73.4, 73.0, 71.8, 71.7, 71.6, 71.5, 71.1, 71.0, 55.7, 51.7.

##### 4b

The crude mixture was purified by column chromatography (silica) with hexane/EtOAc 9:1 and hexane/EtOAc 4:1 to give the product as a pale yellow oil (1.52 g, 2.44 mmol, 73% yield). Rf 0.55 (hexane/EtOAc 1:1, silica); ${[\alpha]}_{D}^{26}$ -22.2˚ (*c* 0.36, CHCl_3_); IR (4000–400 cm^-1^): 3075 w, 3052 m (aromatic C-H), 2916 s with left shoulder (alkyl C-H), 2868 s (alkyl C-H), 2101 s (N_3_), 1559 m, 1497 and 1454 s (aromatic C=C), 1355 s, 1284 s br., 1249 m, 1200 m, 1101 s (C-O), 1059 s (C-O), 1028 s (C-O), 942 m, 852 w, 822 w, 736 and 698 s (aromatic C-H), 609 w, 556 w, 461 w; LRMS (ESI+): m/z calculated 644.29 ([M+Na]^+^), found 644.09 ([M+Na]^+^); ^1^H NMR (400 MHz, MeOD): δ 7.41-7.26 (m, 15H, 15 x aromatic CH), 4.89 (s, 1H, CH), 4.78 (dd, *J* 40.8, 11.6 Hz, 2H, CH_2_), 4.63-4.53 (m, 4H, 2 x CH_2_), 4.28 (dd, *J* 8.8, 4.8 Hz, 1H, CH), 4.12-4.06 (m, 2H, 2 x CH), 3.99 (dd, *J* 10.4, 1.6 Hz, 1H, CH (glucose C6)), 3.75-3.69 (m, 3H, CH and CH_2_ (PEG)), 3.66-3.59 (m, 10H, 5 x CH_2_ (PEG)), 3.35 (s, 3H, CH_3_), 2.90 (s, 1H, CH); ^13^C NMR (100 MHz, MeOD): δ 138.0, 137.6, 137.1, 126.7, 126.7, 126.6, 126.2, 125.9, 125.9, 125.9, 125.8, 125.6, 107.1, 84.5, 79.4, 78.8, 75.3, 71.4, 70.5, 70.3, 69.9, 69.1, 68.9, 68.8, 68.4, 67.7, 56.5, 53.1.

## Methyl 2-(2-[2-(2-aminoethoxy)ethoxy]ethoxy)-3,5,6-tri-O-benzyl-α-D-glucofuranoside (5a) and methyl 2-(2-[2-(2-aminoethoxy)ethoxy]ethoxy)-3,5,6-tri-O-benzyl-β-D-glucofuranoside (5b)

A solution of **4a** or **4b** (1.60 g, 2.57 mmol) in MeOH (25 mL) was stirred under N_2_. Pd(10%)/C (160 mg, 10% w/w) was added and the mixture was stirred under an atmosphere of hydrogen for 2 h at room temperature. The catalyst was removed by filtration through celite and solvent removed from the filtrate to yield the products as a pale-yellow oils (**5a**: 1.47 g, 2.47 mmol, 96% yield; **5b**: 1.50 g, 2.52 mmol, 98% yield).

##### 5a

${[\alpha]}_{D}^{26}$ +76.7˚ (*c* 0.20, CHCl_3_); IR (4000–400 cm^-1^): 3395 w br. (amine N-H), 3063 w, 3031 m (aromatic C-H), 2866 s with left shoulder (alkyl C-H), 1587 m, 1497 and 1454 s (aromatic C=C), 1355 s, 1309 m, 1249 m (C-N), 1193 m, 1098 s (C-O), 1057 s (C-O), 1027 s (C-O), 953 w, 904 w, 818 w, 774 w, 735 and 697 s (aromatic C-H), 606 w, 461 w; LRMS (ESI+): m/z calculated 596.32 ([M+H]^+^), found 596.00 ([M+H]^+^);^1^H NMR (400 MHz, MeOD): δ 7.32-7.24 (m, 15H, 15 x aromatic CH), 5.03 (d, *J* 4.2 Hz, 1H, CH), 4.69 (dd, *J* 11.7, 6.9 Hz, 2H, CH_2_), 4.51 (m, 4H, 2 x CH_2_), 4.29 (dd, *J* 7.2, 5.8 Hz, 1H, CH), 4.14 (dd, *J* 5.7, 3.6 Hz, 1H, CH), 4.04 (t, *J* 4.0 Hz, 1H, CH), 3.97 (ddd, *J* 7.4, 5.4, 2.1 Hz, 1H, CH), 3.86 (dd, *J* 10.8, 2.1 Hz, 1H, CH), 3.74-3.60 (m, 9H, 4 x CH_2_ (PEG) and 1 x CH), 3.58 (t, *J* 5.2 Hz, 2H, CH_2_ (PEG)), 3.40 (s, 3H, CH_3_), 3.35 (s, 2H, NH_2_), 2.90 (t, *J* 5.2 Hz, 2H, CH_2_ (PEG)); ^13^C NMR (100 MHz, MeOD): δ 140.1, 139.8, 139.5, 129.4, 129.3, 129.2, 128.9, 128.7, 128.7, 128.7, 128.5, 128.4, 102.9, 86.2, 83.1, 78.0, 77.9, 74.3, 73.5, 73.3, 73.0, 71.6, 71.5, 71.3, 71.2, 71.0, 55.7, 42.1.

##### 5b

${[\alpha]}_{D}^{26}$ -20.9˚ (*c* 0.86, CHCl_3_); IR (4000–400 cm^-1^): 3392 w br. (amine N-H), 3063 w, 3030 m (aromatic C-H), 2865 s with left shoulder (alkyl C-H), 1585 m, 1497 and 1454 s (aromatic C=C), 1354 s, 1309 m, 1247 m (C-N), 1200 m, 1100 s (C-O), 1059 s (C-O), 1028 s (C-O), 944 w, 818 m, 736 and 697 s (aromatic C-H), 608 w, 462 w; LRMS (ESI+): m/z calculated 596.32 ([M+H]^+^), found 596.28 ([M+H]^+^); ^1^H NMR (400 MHz, MeOD): δ 7.33-7.22 (m, 15H, 15 x aromatic CH), 4.86 (s, 1H, CH), 4.66 (dd, *J* 31.6, 11.6 Hz, 2H, CH_2_), 4.52-4.45 (m, 4H, 2 x CH_2_), 4.28 (dd, *J* 8.0, 4.4 Hz, 1H, CH), 4.04-4.00 (m, 2H, 2 x CH), 3.96 (s, 1H, CH), 3.88 (dd, *J* 10.8, 2.0 Hz, 1H, CH), 3.67 (dd, *J* 10.4, 5.2 Hz, 1H, CH), 3.69-3.65 (m, 8H, 4 x CH_2_ (PEG)), 3.42 (t, *J* 5.2 Hz, 2H, CH_2_ (PEG)), 3.35 (s, 3H, CH_3_), 3.33 (s, 2H, NH_2_), 2.69 (t, *J* 5.6 Hz, 2H, CH_2_ (PEG)); ^13^C NMR (100 MHz, MeOD): δ 140.1, 139.8, 139.3, 129.3, 129.3, 129.2, 129.0, 128.7, 128.7, 0128.6, 128.5, 128.4, 109.6, 87.2, 81.8, 81.4, 77.9, 74.2, 73.4, 73.2, 72.8, 71.6, 71.5, 71.1, 70.8, 70.3, 55.9, 42.0.

## Methyl 2-(2-[2-(2-(tert-butoxycarboxamido)ethoxy)ethoxy] ethoxy)-3,5,6-tri-O-benzyl-α-D-glucofuranoside (6a) and methyl 2-(2-[2-(2-(tert-butoxycarboxamido)ethoxy)ethoxy] ethoxy)-3,5,6-tri-O-benzyl-β-D-glucofuranoside (6b)

**5a** or **5b** (0.70 g, 1.18 mmol) was dissolved in MeCN (17.5 mL). Et_3_N (0.17 mL, 1.18 mmol, 1 eq.) and di-*tert*-butyl dicarbonate (0.26 g, 1.18 mmol, 1 eq.) were added and the reaction mixture stirred for 5 h at room temperature. The solvent was removed and the resulting solid partitioned between H_2_O (10 mL) and EtOAc (10 mL). The EtOAc layer was collected and washed with H_2_O (7 mL). The organic layer was dried over Na_2_SO_4_ and the solvent removed to give the products as yellow oils (**6a**: 0.62 g, 0.89 mmol, 75% yield; **6b**: 0.58 g, 0.83 mmol, 70% yield).

##### 6a

Rf 0.85 (EtOAc, silica); ${[\alpha]}_{D}^{26}$ +34.2˚ (*c* 0.37, CHCl_3_); IR (4000–400 cm^-1^): 3363 w br. (N-H), 3031 w (aromatic C-H), 2869 s with left shoulder (alkyl C-H), 1694 s with left shoulder (C=O), 1520 m (Amide II), 1498 m and 1454 s (aromatic C=C), 1392 m, 1365 s, 1276 m, 1250 m (C-N) , 1101 s (C-O), 1059 m (C-O), 1027 m (C-O), 863 w, 775 w, 736 and 698 s (aromatic C-H), 464 w br., 404 w; LRMS (ESI+): m/z calculated 718.36 ([M+Na]^+^), found 718.00 ([M+Na]^+^); ^1^H NMR (400 MHz, MeOD): δ 7.31-7.24 (m, 15H, 15 x aromatic CH), 5.02 (d, *J* 4.0 Hz, 1H, CH), 4.70 (dd, *J* 11.6, 4.8 Hz, 2H, CH_2_), 4.50 (dt, *J* 2.0, 6.0 Hz, 4H, 2 x CH_2_), 4.29 (dd, *J* 6.8, 6.0 Hz, 1H, CH), 4.15 (dd, *J* 5.6, 3.6 Hz, 1H, CH), 4.05 (t, *J* 4.0 Hz, 1H, CH), 3.97 (ddd, *J* 10.8, 5.6, 2.0 Hz, 1H, CH (glucose C6)), 3.84 (dd, *J* 10.8, 2.0 Hz, 1H, CH (glucose C6)), 3.74-3.55 (m, 10H, 4 x CH_2_ (PEG), 1 x CH and NH), 3.45 (t, *J* 5.6 Hz, 2H, CH_2_ (PEG)), 3.38 (s, 3H, CH_3_), 3.17 (t, *J* 5.6 Hz, 2H, CH_2_ (PEG)), 1.42 (s, 9H, 3 x CH_3_ (*^t^*Bu)); ^13^C NMR (100 MHz, MeOD): δ 158.3, 140.1, 139.8, 139.5, 129.4, 129.3, 129.2, 128.9, 128.8, 128.7, 128.7, 128.5, 128.4, 102.9, 86.3, 83.1, 80.0, 77.9, 77.8, 74.3, 73.3, 73.0, 71.7, 71.6, 71.5, 71.3, 71.1, 71.0, 55.7, 41.3, 28.8.

##### 6b

Rf 0.82 (EtOAc, silica); ${[\alpha]}_{D}^{26}$ -17.5˚ (*c* 0.85, CHCl_3_); IR (4000–400 cm^-1^): 3361 w br. (N-H), 3063 w (aromatic C-H), 3030 m, 2866 s with left shoulder (alkyl C-H), 1711 s (C=O), 1517 m (Amide II), 1498 and 1454 s (aromatic C=C), 1391 m, 1365 s, 1273 m, 1248 m (C-N) , 1101 s (C-O), 1060 s (C-O), 1028 m (C-O), 867 w, 818 w, 779 w, 735 and 697 s (aromatic C-H), 608 m, 463 w br., 403 w; LRMS (ESI+): m/z calculated 718.36 ([M+Na]^+^), found 718.37 ([M+Na]^+^); ^1^H NMR (400 MHz, MeOD): δ 7.31-7.22 (m, 15H, 15 x aromatic CH), 4.86 (s, 1H, CH), 4.65 (dd, *J* 32.4, 11.2 Hz, 2H, CH_2_), 4.53-4.46 (m, 4H, 2 x CH_2_), 4.27 (dd, *J* 8.8, 4.8 Hz, 1H, CH), 4.08 (q, *J* 7.2 Hz, 1H, CH), 4.04-3.99 (m, 2H, 2 x CH), 3.95 (s br., 1H, NH), 3.89 (dd, *J* 10.8, 2.0 Hz, 1H, CH (glucose C6)), 3.66 (dd, *J* 10.8, 5.2 Hz, 1H, CH), 3.60-3.52 (m, 8H, 4 x CH_2_ (PEG)), 3.44 (t, *J* 5.6 Hz, 2H, CH_2_ (PEG)), 3.33 (s, 3H, CH_3_), 3.17 (t, *J* 5.6 Hz, 2H, CH_2_ (PEG)), 1.41 (s, 9H, 3 x CH_3_ (*^t^*Bu)); ^13^C NMR (100 MHz, MeOD): δ 158.3, 140.2, 139.8, 139.4, 129.3, 129.3, 129.2, 129.0, 128.8, 128.7, 128.7, 128.5, 128.4, 109.7, 87.3, 81.9, 81.4, 77.9, 74.3, 73.3, 72.9, 71.7, 71.6, 71.5, 71.2, 71.0, 70.3, 61.45, 55.9, 41.3, 28.8.

## Methyl 2-(2-[2-(2-(tert-butoxycarboxamido)ethoxy)ethoxy] ethoxy)-α-D-glucofuranoside (7a) and methyl 2-(2-[2-(2-(tert-butoxycarboxamido)ethoxy)ethoxy] ethoxy)-β-D-glucofuranoside (7b)

A 9 mM solution of **6a** or **6b** in EtOH was passed through a ThalesNano H-Cube Pro^TM^ flow hydrogenation reactor at 0.3 mL min^-1^. Benzyl ether hydrogenolysis was achieved using a Pd(10%)/C CatCart® at 80 bar and 60 ˚C. The product solution was evaporated to dryness to yield the product as a colourless oil, without any further purification (quantitative yield).

##### 7a

${[\alpha]}_{D}^{26}$ +64.0˚ (*c* 0.50, H_2_O); IR (4000–400 cm^-1^): 3379 s br. (O-H and N-H), 2926 s with right shoulder (alkyl C-H), 1689 s (C=O), 1528 m (Amide II), 1454 m, 1413 w, 1393 w, 1366 s, 1284 m, 1250 s (C-N) , 1092 s with left shoulder (C-O), 1021 m (C-O), 883 m, 861 w, 774 w, 507 s br; LRMS (ESI+): m/z calculated 426.23 ([M+H]^+^), found 426.51 ([M+H]^+^); ^1^H NMR (400 MHz, MeOD): δ 4.99 (d, *J* 4.3 Hz, 1H, CH), 4.37 (dd, *J* 6.0, 5.1 Hz, 1H, CH), 4.19 (dd, *J* 7.1, 6.3 Hz, 1H, CH), 3.88-3.84 (m, 2H, 2 x CH), 3.78-3.73 (m, 3H, 2 x CH and NH), 3.67-3.60 (m, 8H, 4 x CH_2_ (PEG)), 3.51 (t, *J* 5.6 Hz, 2H, CH_2_ (PEG)), 3.39 (s, 3H, CH_3_), 3.22 (t, *J* 5.6 Hz, 2H, CH_2_ (PEG)), 1.44 (s, 9H, 3 x CH_3_ (*^t^*Bu)); ^13^C NMR (100 MHz, MeOD): δ 158.5, 102.7, 87.8, 80.0, 78.4, 76.1, 72.3, 71.6, 71.5, 71.3, 71.2, 71.0, 64.5, 55.5, 41.3, 28.8.

##### 7b

${[\alpha]}_{D}^{26}$ -33.3˚ (*c* 0.24, H_2_O); IR (4000–400 cm^-1^): 3376 s br. (O-H and N-H), 2925 s with left shoulder (alkyl C-H), 2874 s (alkyl C-H), 1690 s (C=O), 1521 m (Amide II), 1454 m, 1391 w, 1366 s, 1279 m, 1249 s (C-N) , 1096 s with left shoulder (C-O), 1041 m (C-O), 943 m (C-O), 866 m, 817 w, 779 w, 568 s br.; LRMS (ESI+): m/z calculated 448.22 ([M+Na]^+^), found 448.21 ([M+Na]^+^); ^1^H NMR (400 MHz, MeOD): δ 4.85 (s, 1H, CH), 4.27 (d, *J* 4.4 Hz, 1H, CH), 4.03 (dd, *J* 8.4, 4.8 Hz, 1H, CH), 3.95-3.91 (m, 1H, CH), 3.83 (s br., 1H, NH), 3.80 (dd, *J* 11.6, 3.2 Hz, 1H, CH), 3.73-3.71 (m, 2H, 2 x CH), 3.65-3.60 (m, 8H, 4 x CH_2_ (PEG)), 3.51 (t, *J* 5.6 Hz, 2H, CH_2_ (PEG)), 3.36 (s, 3H, CH_3_), 3.22 (t, *J* 5.6 Hz, 2H, CH_2_ (PEG)), 1.44 (s, 9H, 3 x CH_3_ (*^t^*Bu)); ^13^C NMR (100 MHz, MeOD): δ 158.4, 109.2, 90.4, 82.6, 80.1, 75.0, 71.8, 71.6, 71.6, 71.2, 71.1, 70.5, 65.2, 55.7, 41.4, 28.8.

## Hydrochloride salt of 2-(2-[2-(2-aminoethoxy)ethoxy]ethoxy)-D-glucose (8)

**7a** or **7b** (0.20 g, 0.47 mmol) was heated at reflux in 0.5 M hydrochloric acid (12 mL) for 24 h. The solvent was removed, and the product was obtained as a beige solid after freeze-drying (0.15 g, 0.47 mmol, quantitative yield). ${[\alpha]}_{D}^{26}$ +43.9˚ (*c* 0.94, H_2_O); IR (4000–400 cm^-1^): 3252 s br. (O-H and N-H), 2920 s with right shoulder (alkyl C-H), 1628 m, 1455 m, 1352 m, 1249 m (C-N) , 1065 s (C-O), 1021 s (C-O), 873 w, 766 w, 516 s br.; LRMS (ESI+): m/z calculated 312.17 ([M+H]^+^) and 334.15 ([M+Na]^+^), found 312.14 ([M+H]^+^) and 334.13 ([M+Na]^+^); HRMS (ESI+): m/z calculated 312.16559 ([M+H]^+^) for C_12_H_26_O_8_N, found 312.16529 ([M+H]^+^); ^1^H NMR (500 MHz, D_2_O): δ 5.46 (d, *J* 3.5 Hz, 0.5H, CH (glucose C1 α-anomer)), 5.18 (br. s, 0.5H, CH (glucose C1 β-anomer)), 3.93-3.84 (m, 3H, 3 x CH), 3.81-3.80 (m, 3H, CH_2_ (PEG) and CH), 3.77 (br. s, 8H, 4 x CH_2_ (PEG)), 3.49-3.36 (m, 2H, 2 x CH), 3.25 (app. t, 2H, CH_2_ (PEG)); ^13^C NMR (100 MHz, D_2_O): δ 95.8, 95.7, 89.8, 82.8, 79.7, 79.6, 75.9, 75.3, 72.2, 72.1, 72.0, 71.2, 70.1, 70.0, 69.9, 96.6, 69.5, 69.2, 66.4, 65.3, 60.7, 60.5, 46.8, 39.1.

## 2-(2-[2-(2-(N-(7-Nitrobenz-2-oxa-1,3-diazol-4-yl))aminoethoxy)ethoxy]ethoxy)-D-glucose (9)

**8** (90 mg, 0.259 mmol, 1.1 eq.) was dissolved in MeOH (3 mL) and Et_3_N (99 µL, 0.706 mmol, 3 eq.) was added. After stirring at 30 ºC for 1 h, NBD-Cl (47 mg, 0.235 mmol, 1 eq.) was added. The reaction was stirred overnight in the dark and at 30 ºC. Solvent was removed and the resulting solid was dissolved in EtOAc (5 mL). Insoluble material was removed by filtration, and the filtrate was dried *in vacuo*. The resulting solid was dissolved in H_2_O (5 mL) and purified on a sephadex LH‑20 column, eluting with H_2_O to give the product as an orange solid (35 mg, 0.074 mmol, 29 % yield).

IR (4000–400 cm^-1^): 3307 s br. (O-H and N-H), 3079 m (aromatic C-H), 2902 s br. (alkyl C-H), 1620 m, 1587 s (N-O), 1530 m (aromatic C=C), 1494 m (aromatic C=C), 1433 m, 1407 m (aromatic C=C), 1302 s (N-O), 1259 m (C-N), 1192 m (C-O), 1079 s (C-O), 1037 s (C-O), 597 m, 481 m br.; LRMS (ESI+): m/z calculated 497.12 ([M+Na]^+^), found 497.15 ([M+Na]^+^); HRMS (ESI+): m/z calculated 497.14903 ([M+Na]^+^) for C_18_H_26_N_4_O_11_Na, found 497.14954 ([M+Na]^+^); ^1^H NMR (400 MHz, D_2_O): δ 8.60 (app. t, 1H, aromatic CH), 6.49 (d, *J* 9.2 Hz, 1H, aromatic CH), 5.41 (d, *J* 3.6 Hz, 0.5H, CH (glucose C1 α-anomer)), 4.66 (d, *J* 8.0 Hz, 0.5H, CH (glucose C1 β-anomer)), 3.96-3.90 (m, 2H), 3.90-3.83 (m, 3H), 3.80-3.78 (m, 3H), 3.76-3.70 (m, 5H), 3.56-3.50 (m, 1H), 3.45-3.35 (m, 2H), 3.34 (dd, *J* 10.0, 3.6 Hz, 1H, CH), 3.08 (t, *J* 8.8 Hz, 1H, CH); ^13^C NMR (100 MHz, D_2_O): δ 197.9, 194.4, 180.3, 169.0, 166.4, 154.9, 141.2, 139.1, 135.4, 127.1, 95.8, 89.8, 82.8, 79.8, 75.8, 75.3, 72.0, 71.3, 71.2, 70.1, 70.0, 70.0, 69.7, 69.7, 69.6, 69.6, 69.5, 69.4, 69.2, 68.3, 68.2, 68.2, 68.1, 60.7, 60.6, 43.2.

## ((N-(7-Nitrobenz-2-oxa-1,3-diazol-4-yl)aminoethoxy)ethoxy)ethanol (10)

A solution of 2-[2-(2-aminoethoxy)ethoxy]ethanol (0.41 g, 2.75 mmol, 1.1 eq.) and Et_3_N (660 µL, 4.75 mmol, 1.9 eq.) in methanol (15 mL) was stirred at 30°C for 1 h. NBD-Cl (0.5 g, 2.50 mmol, 1 eq.) was added and the reaction was stirred in the dark for 16 h at 30°C. Insoluble material was removed by filtration, and the filtrate was dried. The resulting solid was dissolved in H_2_O (5 mL) and purified on a sephadex LH‑20 column, eluting with H_2_O to give the product as an orange solid (0.33 g, 1.05 mmol, 42% yield).

IR (4000–400 cm^-1^): 3536 m, 3413 s br. (O-H), 3200 w (N-H), 3137 w, 3059 s (aromatic C-H), 2908 s with left shoulder (alkyl C-H), 1621 m (N-H), 1588 s with left shoulder (N-O), 1531 s (aromatic C=C), 1494 s (aromatic C=C), 1437 m, 1412 m (aromatic C=C), 1352 m, 1324 s (N‑O), 1296 m, 1269 m (C-N), 1237 s, 1168 w, 1106 s (C-O), 1085 s (C-O), 1061 s (C-O), 995 s, 824 m, 738 m, 594 m, 515 w br.; LRMS (ESI-): m/z calculated 311.10 ([M-H]^-^), found 311.18 ([M-H]^-^), (ESI+): m/z calculated 335.27 ([M+Na]^+^), found 335.09 ([M+Na]^+^); HRMS (ESI+): m/z calculated 335.09621 ([M+Na]^+^) for C_12_H_16_N_4_O_6_Na, found 335.09650 ([M+Na]^+^); ^1^H NMR (400 MHz, D_2_O): δ 8.63 (d, *J* 9.2 Hz, 1H, aromatic CH), 6.51 (d, *J* 9.2 Hz, 1H, aromatic CH), 3.96-3.94 (m, 2H, CH_2_), 3.88 (br. s, 2H, CH_2_), 3.80-3.77 (m, 2H, CH_2_), 3.72-3.68 (m, 4H, 2xCH_2_), 3.64-3.62 (m, 2H, CH_2_), 1.35 (d, *J* 6.8 Hz, 1H, OH) ^13^C NMR (100 MHz, D_2_O): δ 190.1, 174.9, 148.3, 146.3, 140.5, 113.1, 71.7, 69.8, 69.6, 69.5, 66.4, 60.3.
