# Supplementary material for: A Warburg effect targeting vector designed to increase the uptake of compounds by cancer cells demonstrates glucose and hypoxia dependent uptake
Source: PLoS One. 2019 Jul 15;14(7):e0217712. doi: 10.1371/journal.pone.0217712 (PMC6629077; doi:10.1371/journal.pone.0217712)
Supplement: S1 Dataset — (DOCX) [file pone.0217712.s005.docx]

A Warburg effect targeting vector designed to increase the uptake of compounds by cancer cells demonstrates glucose and hypoxia dependent uptake

Alexandra Glenister,^§^ Michela I. Simone,^†^ and Trevor W. Hambley^§^

^§^ School of Chemistry, University of Sydney, New South Wales, 2006, Australia

^†^ Discipline of Chemistry, Priority Research Centre for Chemical Biology & Clinical Pharmacology, University of Newcastle, Callaghan, New South Wales, 2308, Australia.

*Supporting Information*

# Raw data for Fig 4

Values used to build graph: Relative fluorescence intensity (± standard deviation)

|  | Glucose Concentration in Media | | | | |
| --- | --- | --- | --- | --- | --- |
| Compound |  | 0 mg L^-1^ | 1000 mg L^-1^ | 4000 mg L^-1^ | 8000 mg L^-1^ |
|  | **2-NBDG** | 100.0 ± 6.4 | 83.4 ± 7.3 | 73.7 ± 5.8 | 63.2 ± 4.2 |
|  | ***9*** | 100.0 ± 9.3 | 81.4 ± 9.1 | 56.2 ± 7.3 | 29.5 ± 6.1 |
|  | ***10*** | 100.0 ± 9.6 | 108.4 ±12.3 | 128.9 ± 11.8 | 121.4 ± 17.8 |

Values behind means and standard deviation: Relative fluorescence intensity for cell regions (30 µm x 30 µm)

|  | Glucose Concentration in Media | | | | |
| --- | --- | --- | --- | --- | --- |
| Compound |  | 0 mg L^-1^ | 1000 mg L^-1^ | 4000 mg L^-1^ | 8000 mg L^-1^ |
|  | **2-NBDG** | 97.9  111.7  98.5  102.3  96.2  102.6  110.0  90.2  100.4  96.8 | 71.4  86.6  82.1  90.9  87.4  73.9  91.6  83.4  76.8  90.2 | 67.5  71.7  69.7  66.3  84.9  75.5  80.6  77.1  72.0  72.1 | 53.8  62.3  67.2  64.0  68.2  59.9  67.6  62.9  62.6  63.8 |
|  | ***9*** | 92.6  87.0  107.6  95.9  88.1  99.1  105.9  102.5  110.8  113.2 | 95.6  88.3  86.3  63.98  84.0  84.4  71.7  83.0  82.2  74.2 | 46.7  46.2  55.1  62.4  57.8  63.3  51.9  55.5  69.4  53.5 | 19.8  34.1  36.4  33.9  32.8  29.4  24.6  35.5  20.9  27.1 |
|  | ***10*** | 114.2  115.6  101.8  101.1  99.7  102.2  97.1  88.4  88.1  90.1 | 123.7  136.1  101.0  99.9  102.7  108.3  110.5  104.1  97.9  99.9 | 144.2  135.5  146.9  115.9  126.8  139.2  117.3  125.7  121.2  116.2 | 109.1  116.2  97.9  143.7  138.6  132.7  100.2  132.8 |

Raw data values of fluorescence intensities for cell regions (30 µm x 30 µm). Fluorescence intensities were determined by quantification with LAS AF Lite.

|  | Glucose Concentration in Media | | | | |
| --- | --- | --- | --- | --- | --- |
| Compound |  | 0 mg L^-1^ | 1000 mg L^-1^ | 4000 mg L^-1^ | 8000 mg L^-1^ |
|  | **2-NBDG** | 2429  2771  2444  2538  2387  2545  2729  2238  2491  2402 | 1772  2149  2037  2255  2169  1834  2273  2069  1906  2238 | 1675  1779  1729  1645  2106  1873  2000  1913  1786  1789 | 1335  1546  1667  1588  1692  1486  1677  1561  1553  1583 |
|  | ***9*** | 402  378  465  410  383  430  459  445  481  491 | 401  371  362  269  353  354  301  348  345  311 | 196  194  231  262  243  266  218  233  291  225 | 83  143  153  142  138  123  103  149  88  114 |
|  | ***10*** | 459  464  409  406  401  411  390  355  354  362 | 497  547  406  401  413  435  444  418  393  401 | 579  544  590  466  509  559  471  505  487  467 | 438  467  393  577  557  533  403  534 |

**Data for S1 Fig**

Values used to build graph: Relative rate of HK phosphorylation (*Vi* (substrate)/*Vi*) at increasing concentrations of compound (substrate)

|  | Compound | | |
| --- | --- | --- | --- |
| Concentration of Compound (µM) | Glucosamine hydrochloride | 2-[2-(2-aminoethoxy)ethoxy]ethanol | ***8*** |
| 36 | 1.00 |  | 0.98 |
| 71 |  | 1.00 | 0.97 |
| 143 | 0.89 | 0.96 | 0.96 |
| 214 |  |  |  |
| 286 | 0.78 |  | 0.90 |
| 357 | 0.79 | 0.97 |  |
| 714 | 0.61 | 0.99 | 0.88 |

# High Resolution Mass Spectra


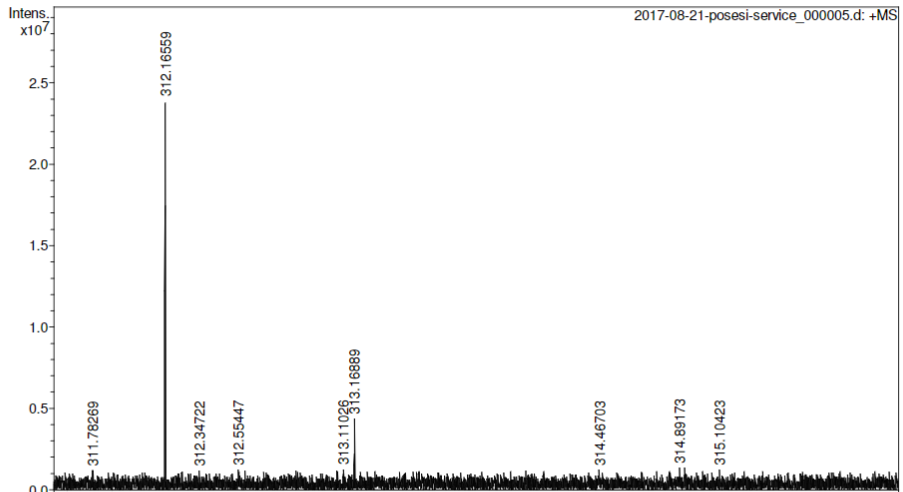


2-(2-[2-(2-aminoethoxy)ethoxy]ethoxy)-D-glucose (***8***)


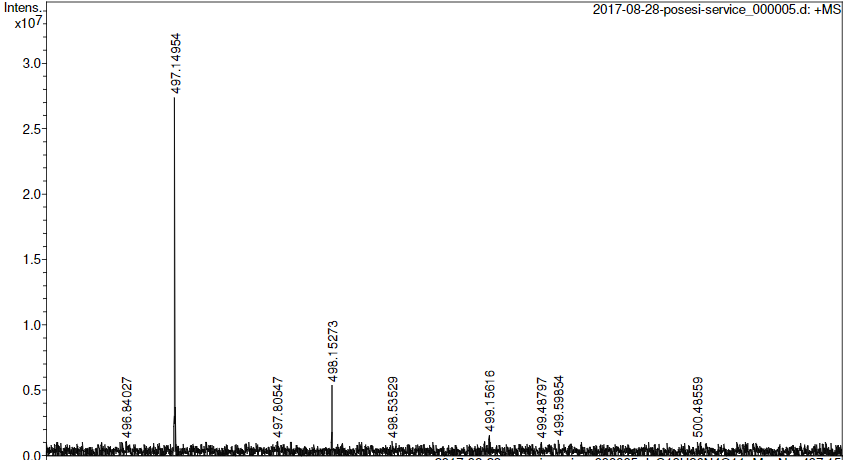


2-(2-[2-(2-(N-(7-Nitrobenz-2-oxa-1,3-diazol-4-yl))aminoethoxy)ethoxy]ethoxy)-D-glucose (**9**)


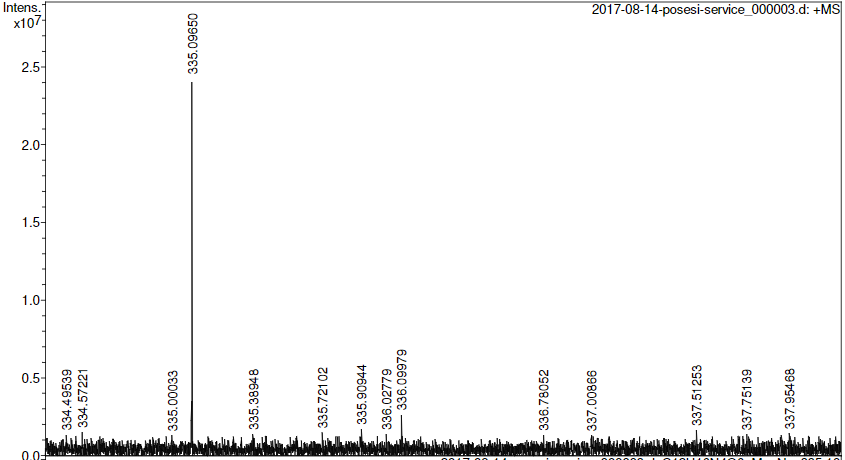


((N-(7-Nitrobenz-2-oxa-1,3-diazol-4-yl)aminoethoxy)ethoxy)ethanol (**10**)

# ^1^H NMR Spectra


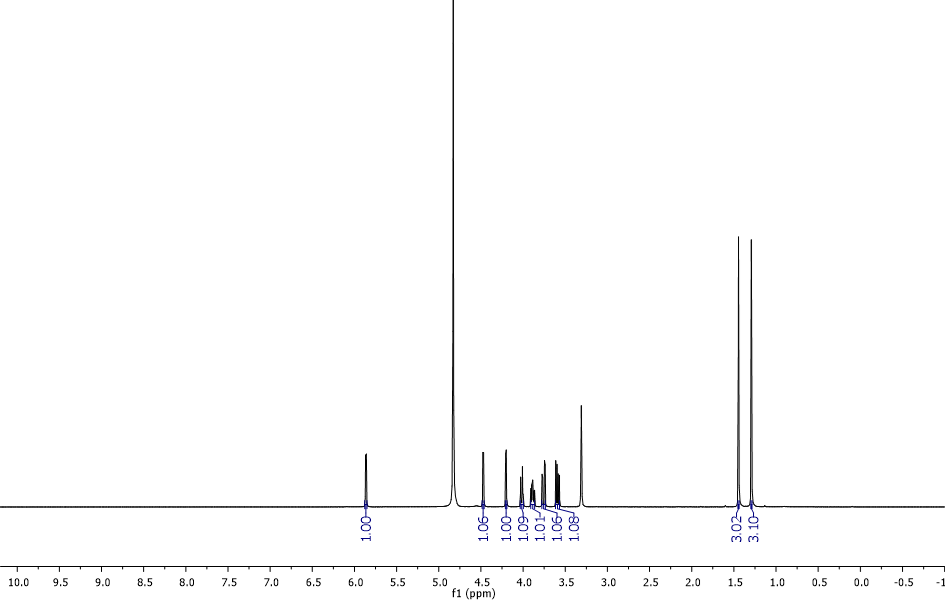


1,2-O-isopropylidene-α-D-glucofuranose (***1***)


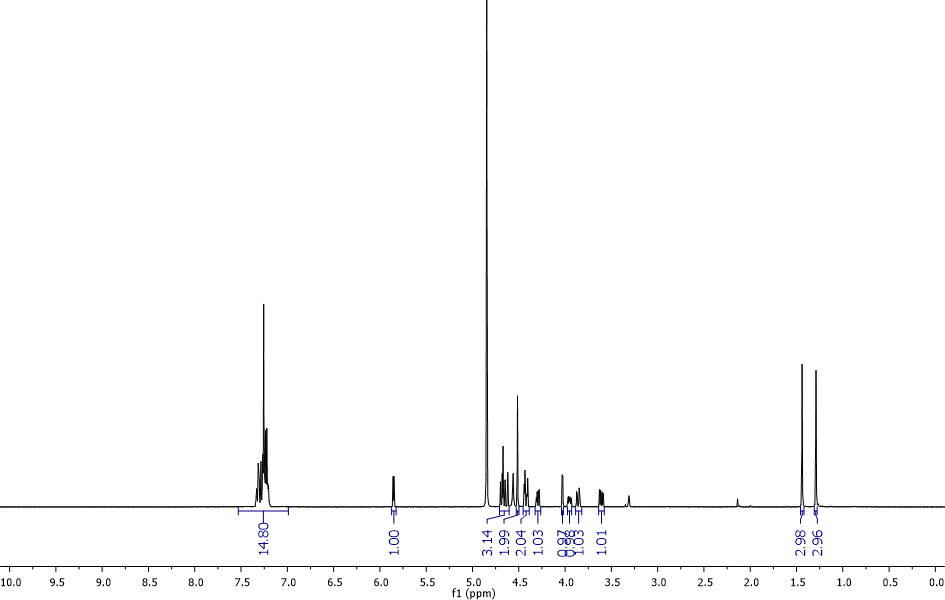


3,5,6-tri-O-benzyl-1,2-O-isopropylidene-α-D-glucofuranose (***2***)


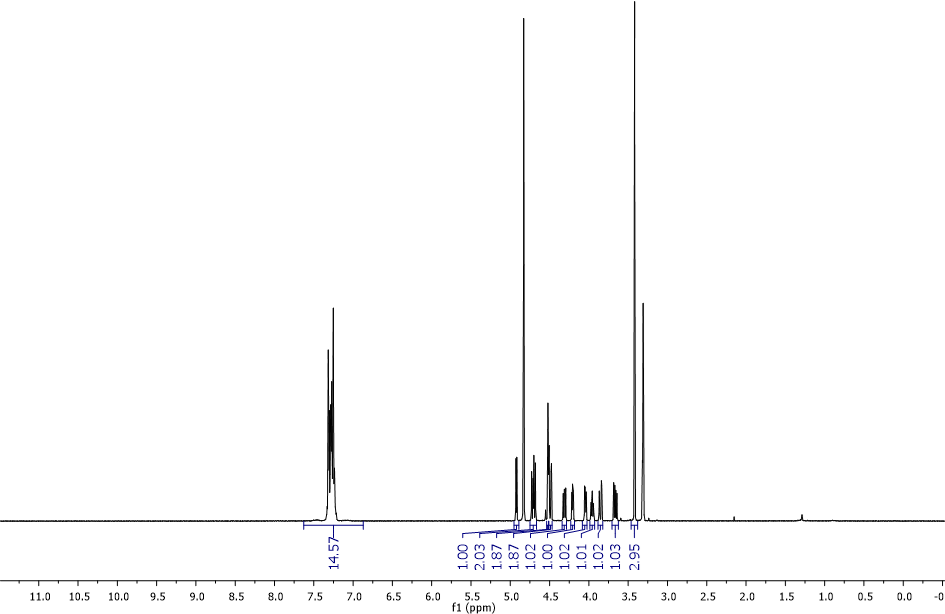


Methyl 3,5,6-tri-O-benzyl-α-D-glucofuranoside (***3a***)


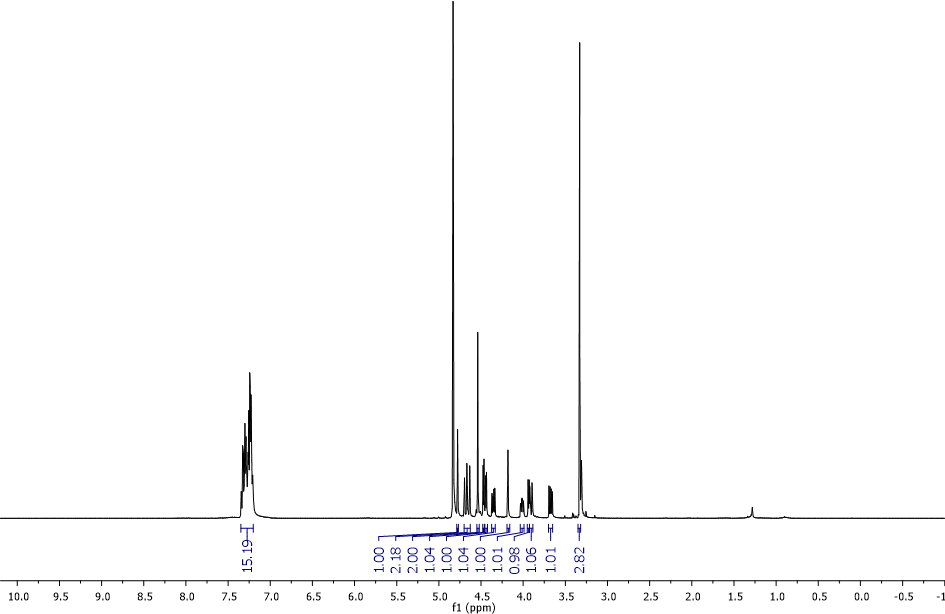


Methyl 3,5,6-tri-O-benzyl-β-D-glucofuranoside (***3b***)


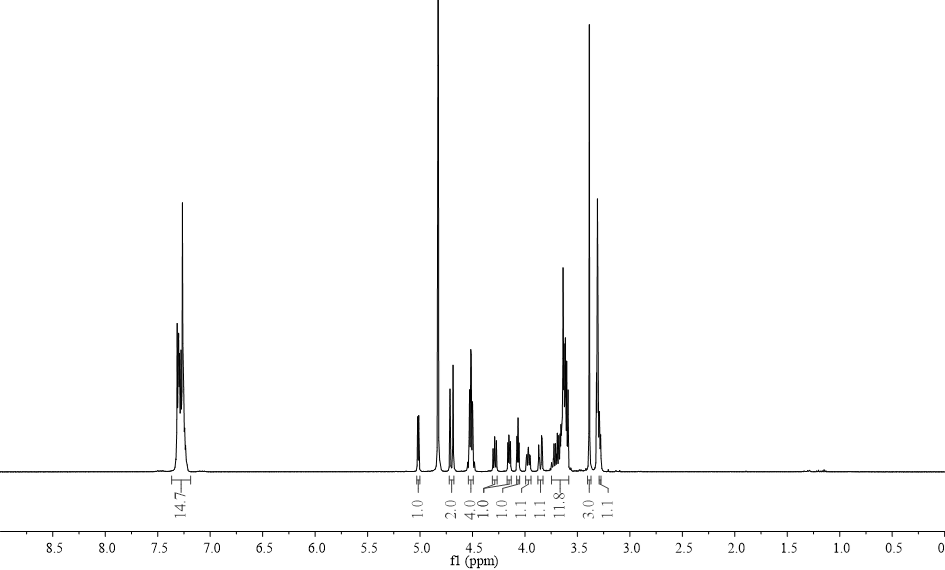


Methyl 2-(2-[2-(2-azidoethoxy)ethoxy]ethoxy)-3,5,6-tri-O-benzyl-α-D-glucofuranoside (***4a***)

Methyl 2-(2-[2-(2-azidoethoxy)ethoxy]ethoxy)-3,5,6-tri-O-benzyl-β-D-glucofuranoside (***4b***)


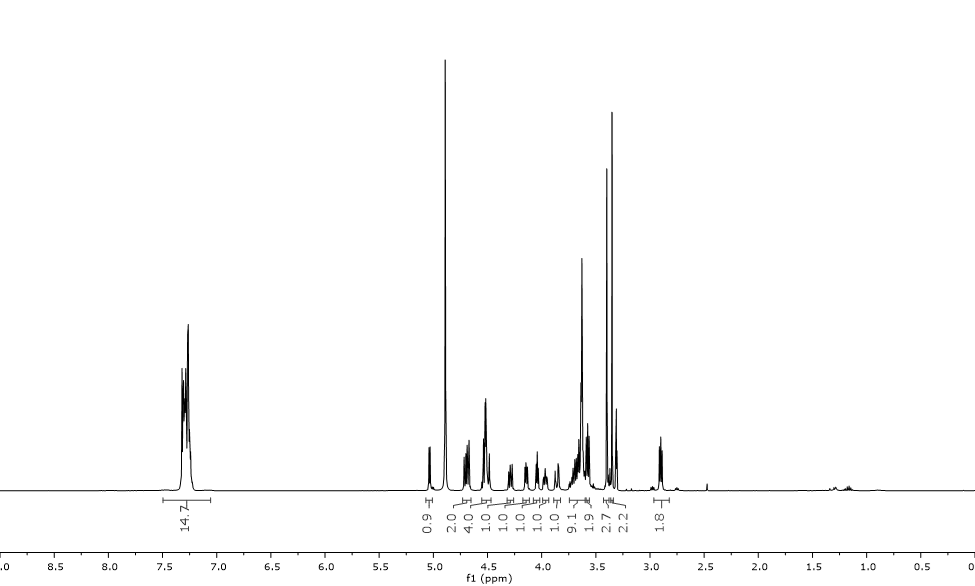


Methyl 2-(2-[2-(2-aminoethoxy)ethoxy]ethoxy)-3,5,6-tri-O-benzyl-α-D-glucofuranoside (***5a***)

Methyl 2-(2-[2-(2-aminoethoxy)ethoxy]ethoxy)-3,5,6-tri-O-benzyl-β-D-glucofuranoside (***5b***)


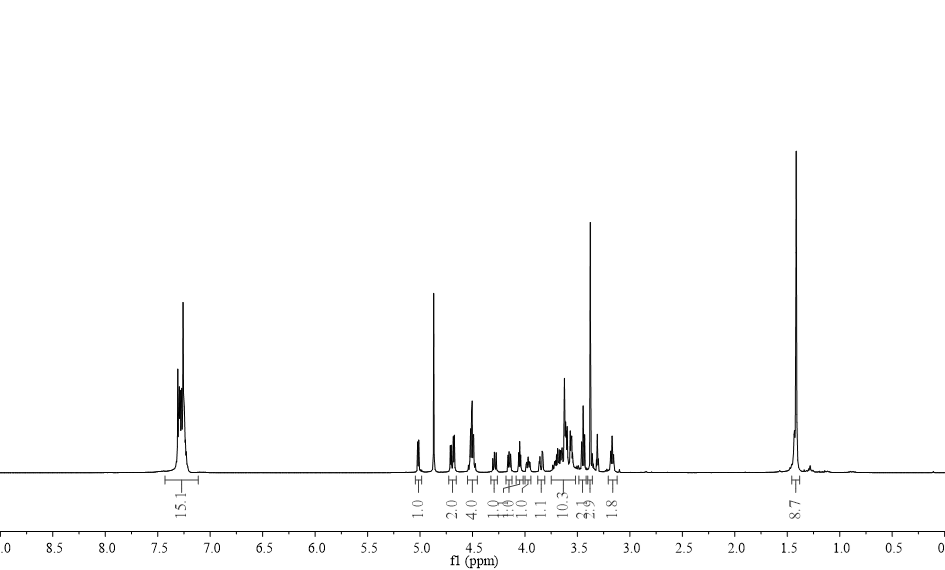


Methyl 2-(2-[2-(2-(tert-butoxycarboxamido)ethoxy)ethoxy]ethoxy)-3,5,6-tri-O-benzyl-α-D-glucofuranoside (***6a***)


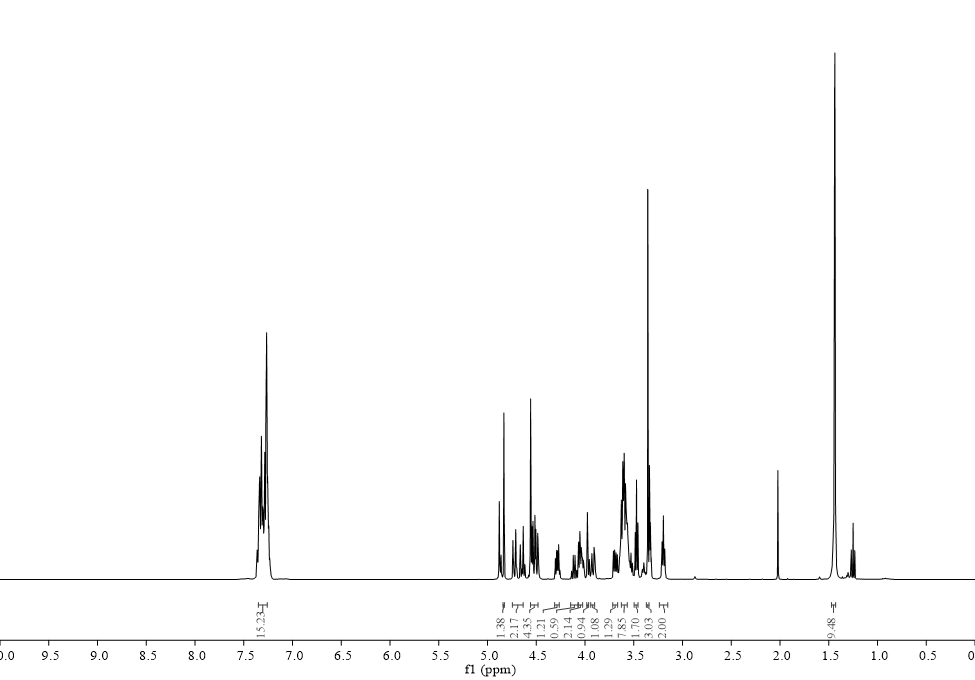


Methyl 2-(2-[2-(2-(tert-butoxycarboxamido)ethoxy)ethoxy]ethoxy)-3,5,6-tri-O-benzyl-β-D-glucofuranoside (***6b***)


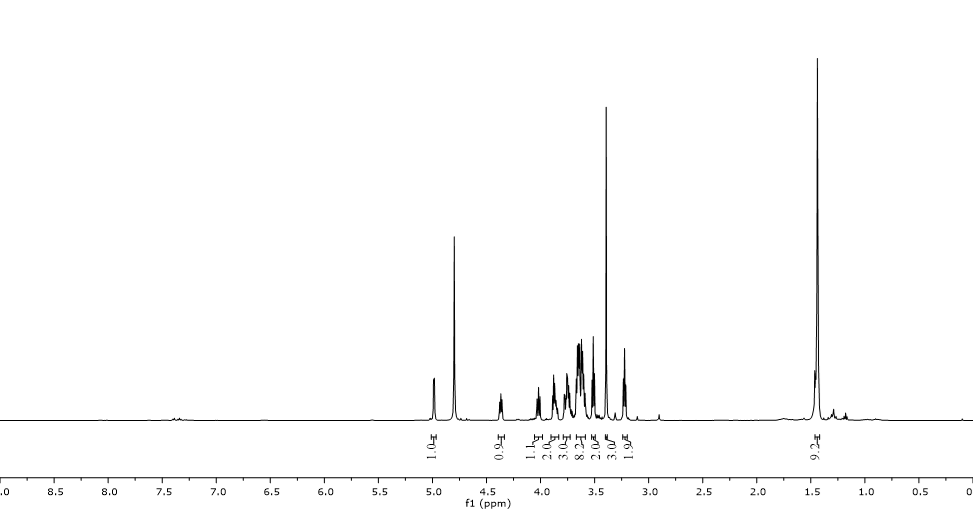


Methyl 2-(2-[2-(2-(tert-butoxycarboxamido)ethoxy)ethoxy]ethoxy)-α-D-glucofuranoside (***7a***)

Methyl 2-(2-[2-(2-(tert-butoxycarboxamido)ethoxy)ethoxy]ethoxy)-β-D-glucofuranoside (***7b***)

# ^13^C NMR Spectra


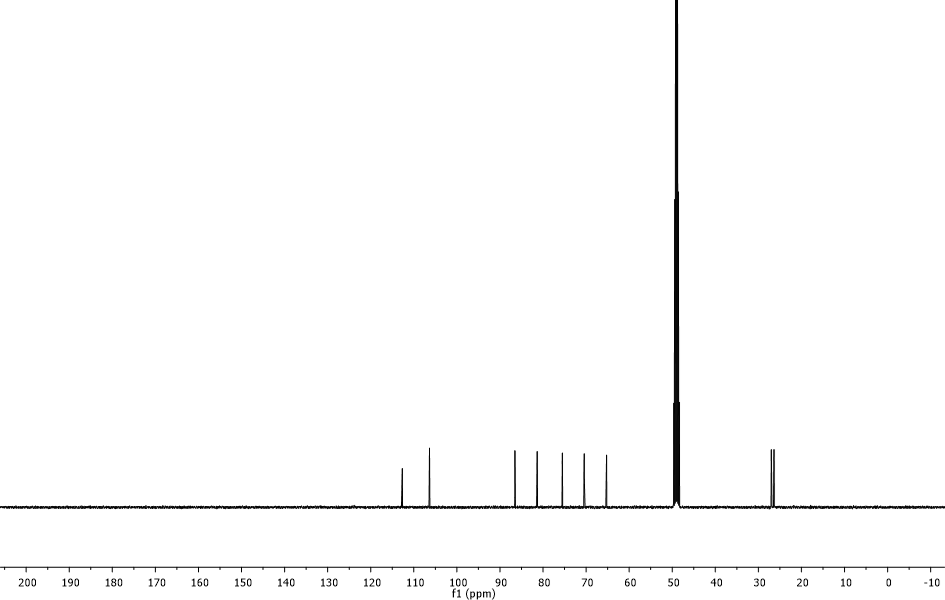


1,2-O-isopropylidene-α-D-glucofuranose (***1***)


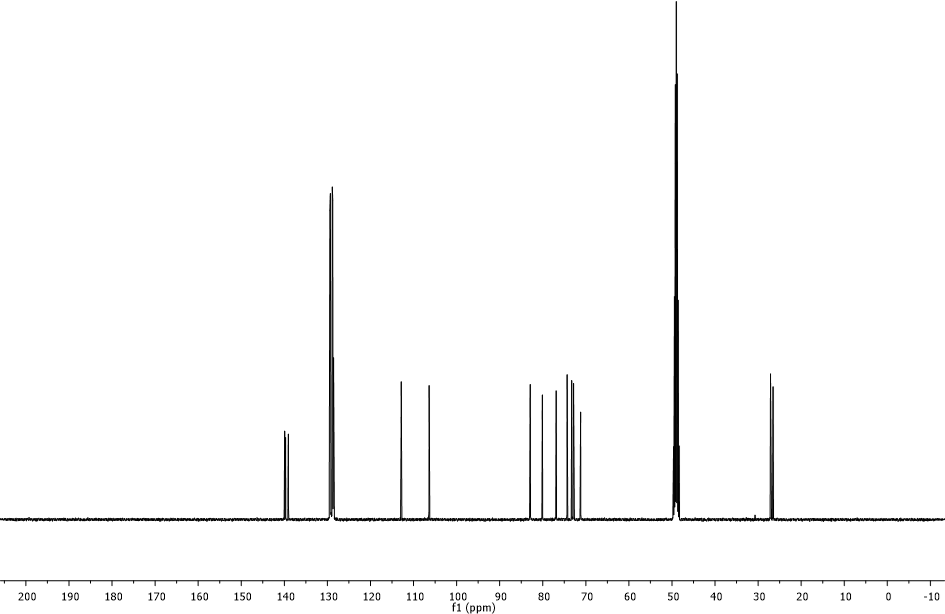


3,5,6-tri-O-benzyl-1,2-O-isopropylidene-α-D-glucofuranose (***2***)


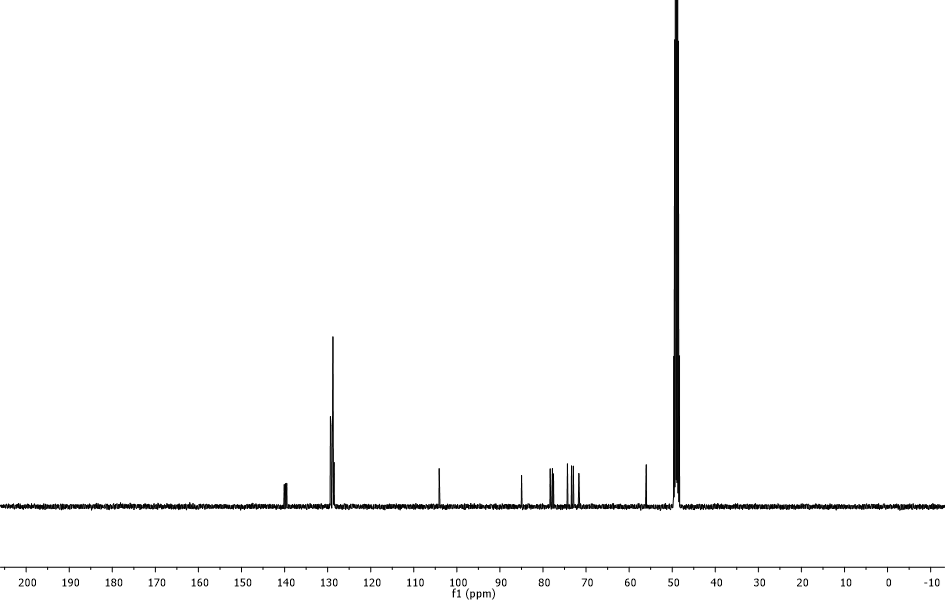


Methyl 3,5,6-tri-O-benzyl-α-D-glucofuranoside (***3a***)


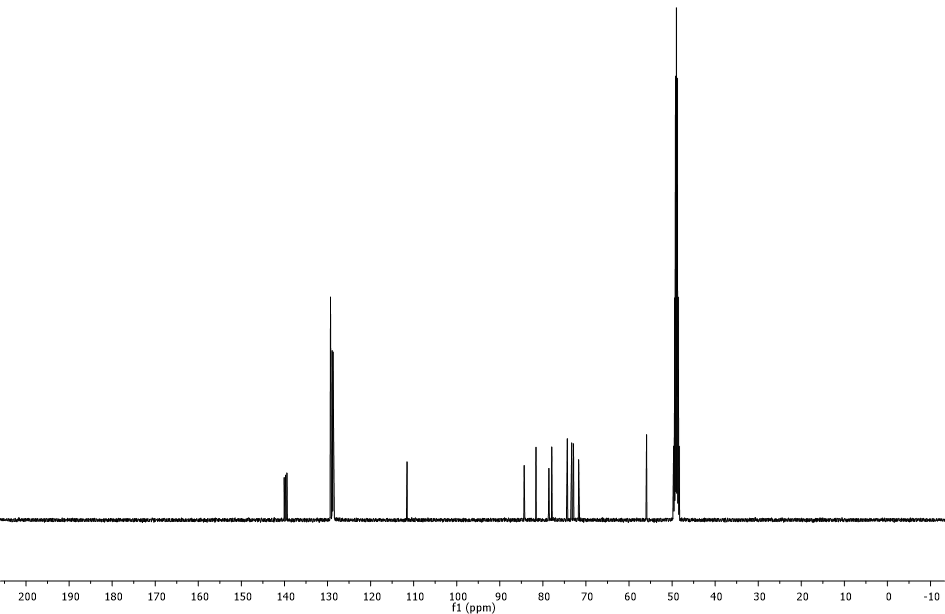


Methyl 3,5,6-tri-O-benzyl-β-D-glucofuranoside (***3b***)


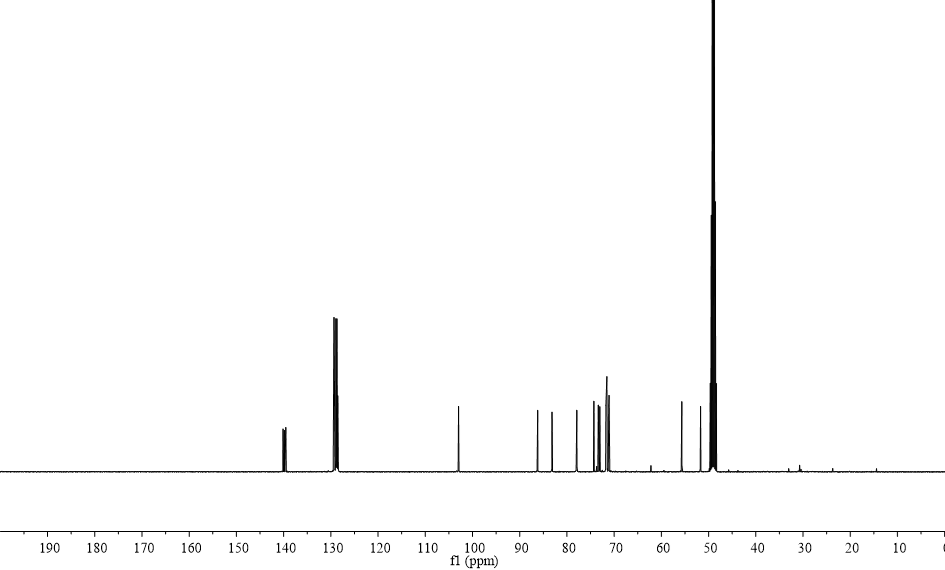


Methyl 2-(2-[2-(2-azidoethoxy)ethoxy]ethoxy)-3,5,6-tri-O-benzyl-α-D-glucofuranoside (***4a***)

Methyl 2-(2-[2-(2-azidoethoxy)ethoxy]ethoxy)-3,5,6-tri-O-benzyl-β-D-glucofuranoside (***4b***)


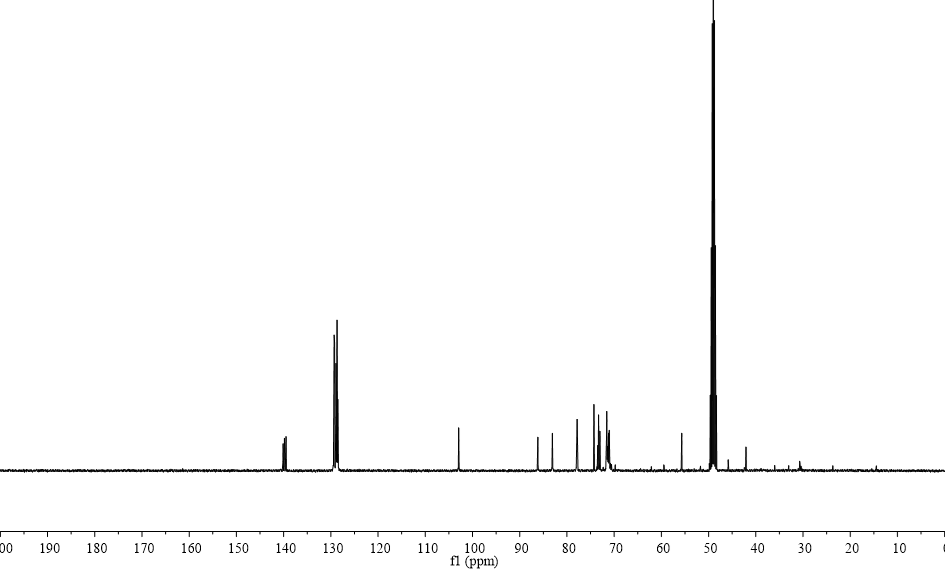


Methyl 2-(2-[2-(2-aminoethoxy)ethoxy]ethoxy)-3,5,6-tri-O-benzyl-α-D-glucofuranoside (***5a***)

Methyl 2-(2-[2-(2-aminoethoxy)ethoxy]ethoxy)-3,5,6-tri-O-benzyl-β-D-glucofuranoside (***5b***)


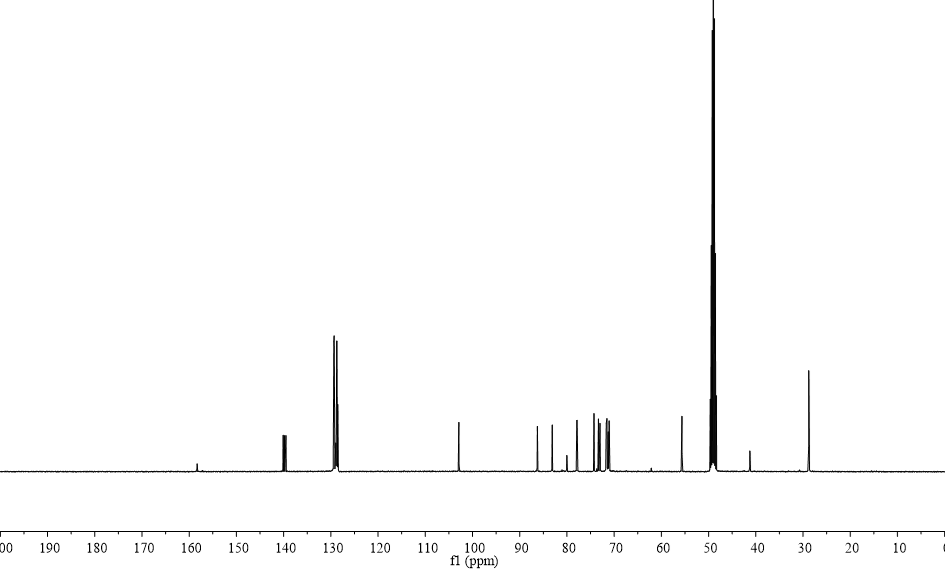


Methyl 2-(2-[2-(2-(tert-butoxycarboxamido)ethoxy)ethoxy]ethoxy)-3,5,6-tri-O-benzyl-α-D-glucofuranoside (***6a***)


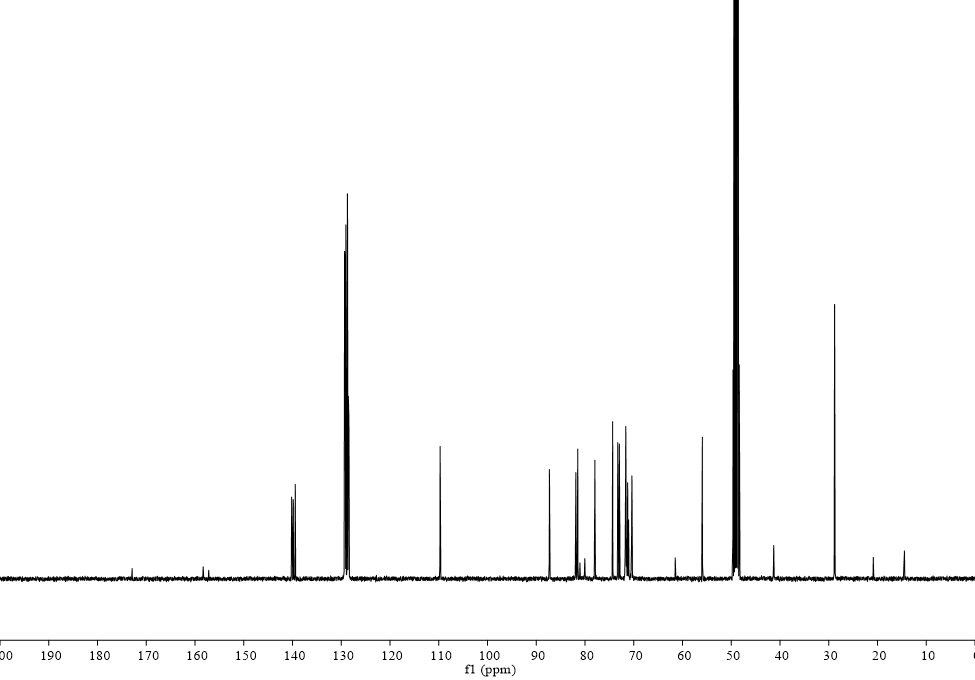


Methyl 2-(2-[2-(2-(tert-butoxycarboxamido)ethoxy)ethoxy]ethoxy)-3,5,6-tri-O-benzyl-β-D-glucofuranoside (***6b***)


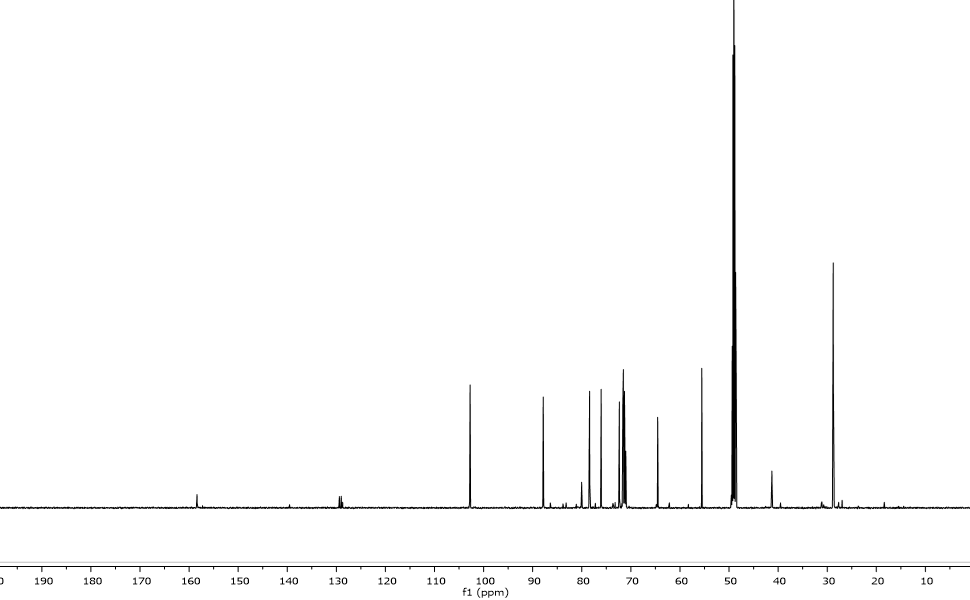


Methyl 2-(2-[2-(2-(tert-butoxycarboxamido)ethoxy)ethoxy]ethoxy)-α-D-glucofuranoside (***7a***)

Methyl 2-(2-[2-(2-(tert-butoxycarboxamido)ethoxy)ethoxy]ethoxy)-β-D-glucofuranoside (***7b***)
